# Spectral and Topological Abnormalities of Resting and Task State EEG in Chinese Children with Developmental Dyslexia

**DOI:** 10.1007/s10548-025-01123-0

**Published:** 2025-06-10

**Authors:** Yaqi Yang, Shuting Huo, Jie Wang, Urs Maurer

**Affiliations:** 1 Department of Psychology, The Chinese University of Hong Kong, Hong Kong, China; 2 Department of Psychology, The Education University of Hong Kong, Hong Kong, China; 3 Centre for Developmental Psychology, The Chinese University of Hong Kong, Hong Kong, China; 4 Brain and Mind Institute, The Chinese University of Hong Kong, Hong Kong, China

**Keywords:** Developmental dyslexia, EEG, Spectral power, Brain topology

## Abstract

Developmental dyslexia (DD) is a common reading disorder with neurological underpinnings; however, it remains unclear whether Chinese children with DD exhibit spectral power or network topology abnormalities. This study investigated spectral power and brain network topology abnormalities using electroencephalography (EEG) during resting states and a one-back Chinese-Korean character task in 85 Hong Kong Chinese children with DD and 51 typically developing peers (ages 7–11). EEG signals were transformed using the Fast Fourier Transform to estimate spectral power. Functional connectivity matrices were derived using the phase-lag index, and network topology was assessed via minimum spanning tree (MST) analysis. The results suggested that children with DD showed reduced alpha power over central, frontal, temporal, parietal, and occipital scalp areas at rest, and over central and frontal areas during the task. MST results revealed decreased beta band integration at rest but increased alpha band integration during the one-back task. Familiar Chinese stimuli elicited greater alpha and beta power and lower beta band integration compared to unfamiliar Korean stimuli. Moreover, resting-state beta band integration correlated positively with reading fluency in children with DD. These findings point to inhibitory control deficits and cortical hyperactivation in Chinese DD, reflected in disrupted large-scale network topology, and highlight the alpha band as a potential biomarker. They also demonstrate that language familiarity modulates neural efficiency and recruits compensatory networks. Overall, the study provides new insights into the neural basis of reading difficulties in Chinese children with DD.

**Supplementary Information:**

The online version contains supplementary material available at 10.1007/s10548-025-01123-0.

## Introduction

Developmental dyslexia (DD) is a widespread neurodevelopmental disorder, affecting approximately 5–10% of children globally, with prevalence estimates varying depending on diagnostic criteria (Pennington and Bishop 2009). It is characterized by persistent deficits in reading, writing, and spelling despite adequate intelligence, educational access, motivation, and intact sensory abilities (Shaywitz and Shaywitz 2005). Neuroimaging studies have consistently identified structural and functional abnormalities in core reading-related brain regions, including the left temporoparietal cortex, left occipitotemporal cortex, and left inferior frontal gyrus—regions implicated in phonological processing, orthographic recognition, and reading fluency (Brem et al. 2020; Hoeft et al. 2007; Richlan et al. 2011). Beyond academic challenges, DD is linked to psychosocial issues such as stress and anxiety, highlighting its significant impact as a learning disability (Eissa 2010; Espin et al. 2019).

### Cognitive Deficits in DD

DD is a multifaceted disorder characterized by a range of cognitive deficits, including impairments in phonological processing (Ramus et al. 2003), orthographic skills (Boros et al. 2016), morphological awareness (Shu et al. 2006), and working memory (Fostick and Revah 2018). The diverse cognitive impairments associated with DD underscore its complexity and suggest the involvement of various neural anomalies underlying the disorder. In alphabetic languages, phonological awareness is traditionally considered the core deficit (Melby-Lervåg and Lervåg 2012). However, in non-alphabetic languages such as Chinese, which lack phoneme-to-grapheme correspondence, deficits in orthographic and morphological awareness are more pronounced (Ho et al. 2007; McBride-Chang et al. 2011; Yan et al. 2013). These linguistic differences suggest both unique and shared cognitive processes across alphabetic and non-alphabetic language systems. Consequently, the underlying neural mechanisms of DD may also differ between these writing systems, reflecting language-specific processing demands.

### Neural Connectivity and Large-Scale Network Abnormalities in DD

Neuroimaging studies have traditionally focused on identifying specific brain regions related to reading. However, it is now recognized that reading requires the intricate coordination of a widespread neural network (Cattinelli et al. 2013). Recent neuroimaging research has shifted focus toward investigating connectivity and large-scale brain network abnormalities in DD. Resting-state fMRI studies indicate widespread connectivity abnormalities. For example, a German study found that children with DD exhibit reduced connectivity between the left posterior and inferior frontal areas (Schurz et al., 2015). Reduced connectivity in the visual word form area and left inferior frontal gyrus has been observed in children with DD during reading tasks (Schurz et al., 2015; Van der Mark et al., 2009). Research on Chinese children with DD has shown decreased connectivity between the left middle occipital gyrus and the left inferior frontal gyrus during lexical processing and visual perception tasks (Cao et al., 2018).

Large-scale brain network abnormalities in children with DD have been investigated using fMRI and brain topology methods during resting and task states (Bailey et al. 2018; Zhang et al. 2021). Resting-state studies suggest that children with DD show lower connectivity clustering in the left hemisphere than their typically developing (TD) peers (Finn et al. 2014; Qi et al. 2016). However, task-based findings have yielded inconsistent results. While some studies have reported over-segregated functional organization in Chinese children with DD during phonological tasks (Zhang et al. 2021), others have found no significant group differences, suggesting task-dependent neural adaptations (Yang and Tan 2020). These discrepancies underscore the need for further research to explore how task demands and linguistic differences influence brain connectivity patterns in DD.

### EEG Spectral and Connectivity Abnormalities in DD

EEG has provided valuable insights into the neurophysiological underpinnings of DD during resting-state and task-state conditions. Specifically, studies on EEG spectral power have revealed reduced alpha band activity (8–12 Hz) across the frontal, temporal, and parietal scalp areas in children with DD (Babiloni et al. 2012; Papagiannopoulou and Lagopoulos 2016). This diminished alpha activity, often localized in the temporoparietal scalp area—crucial for decoding and word recognition—potentially indicates a disruption in the neural mechanisms underlying phonological processing (Angelakis et al. 2004). Furthermore, children with DD have shown decreased beta band activity (12–30 Hz) over the frontal and central scalp areas (Eroğlu et al. 2022; Spironelli et al. 2008). Since beta activity is associated with executive functions and visuomotor integration, its reduction further indicates impairments in broader cognitive processing domains (Basharpoor et al. 2022). Notably, findings from De Vos et al. (2017) suggest that increased beta band power to speech rhythms in adolescents with DD may represent a compensatory mechanism. In their study, adolescents with DD showed reduced alpha synchronization but elevated beta responses, and importantly, beta power positively correlated with phonological performance. These results suggest that beta oscillatory activity may support phonemic processing in the absence of efficient alpha entrainment.

Beyond spectral power, EEG connectivity studies have unveiled that children with DD display reduced connectivity between the occipital and temporal scalp areas, indicating a disruption in the neural circuitry that supports reading (Gallego-Molina et al. 2022; Žarić et al. 2017). Resting-state EEG source analyses have also revealed weaker connectivity among regions involved in visual and motor processing, including the calcarine sulcus, right postcentral gyrus, left paracentral gyrus, right angular gyrus, and right supplementary motor area (Bosch-Bayard et al. 2020). In contrast, other studies have reported increased coherence across widespread scalp regions—particularly frontal, central, and temporal areas—across all frequency bands (delta, theta, alpha, and beta), which may reflect compensatory reorganization of functional networks in response to reading difficulties (Arns et al., 2007).

### Brain Network Integration and Topology Abnormalities in DD

Minimum Spanning Tree (MST) analysis is increasingly used to explore global brain connectivity, offering insights into neural network organization while avoiding the confounds of recurrent connections. MST constructs a cycle-free network that connects all brain regions with minimal total weight, enabling a more efficient assessment of network structure (Stam et al. 2014; Tewarie et al. 2015a). In the context of DD, the application of MST to resting-state EEG data has consistently exposed atypical brain topology. For instance, Fraga González et al. (2016) identified a less integrated theta band network in Dutch children with DD. Similarly, Xue et al. (2020) reported a less integrated theta band network in Chinese children with DD, along with reduced beta band integration, suggesting potential cultural or linguistic specificity in brain network alterations associated with DD.

In contrast, studies in adults with DD have shown more integrated alpha band networks (Fraga González et al. 2018), interpreted as a compensatory reorganization that may reflect increased reliance on alternative or bilateral neural pathways to support reading. Since DD is a neurological disorder of reading (Habib 2000), examining brain topology differences in EEG during reading tasks would be valuable. However, there are few task-state EEG studies investigating brain topology abnormalities. Only a task-state EEG study of Dutch adults with DD during auditory tasks found that individuals with DD exhibit a stronger shift toward a more integrated theta band network topology during word tracking, suggesting altered functional brain network organization linked to impaired phonological and reading skills (Zhang et al. 2022).

### The Current Study

Despite significant advancements, critical gaps in our understanding of DD remain. Research on brain network topology in non-alphabetic languages, such as Chinese, is notably scarce, particularly studies examining spectral power and topological abnormalities. Moreover, existing studies are often limited by small sample sizes (Fraga González et al. 2016; Xue et al. 2020; Zhang et al. 2022), reducing statistical power and generalizability. Additionally, most MST-based investigations have focused exclusively on resting-state data. Given DD’s reading-related nature, examining differences in brain topology during reading tasks may provide more ecologically valid insights. Resting-state activity predominantly reflects aperiodic or spontaneous neural activation, while task-related activity is more directly linked to the specific neural processes engaged by the task and is thus capable of revealing band-specific neural dynamics. To address these limitations, the present study adopts a comprehensive approach, integrating both resting and task states EEG in a large cohort of Chinese children with DD (*n* = 85). This larger sample size enables robust statistical analyses and the investigation of neural impairments in relation to dyslexia severity. This approach is expected to advance the understanding of DD’s neural mechanisms and inform targeted intervention strategies.

Therefore, our study aims to examine brain activity and network topology abnormalities in Hong Kong Chinese children with DD in both resting and task states. EEG recordings were obtained from children with DD and their TD peers during resting-state sessions—with eyes open and closed—and during a Chinese-Korean one-back task. We calculated spectral power for several scalp areas in the alpha and beta bands and constructed functional connectivity matrices using the phase-lag index (PLI). From each matrix, we extracted MST graphs and computed related metrics. Finally, to explore the potential brain-behaviour connection, we also conducted correlational analyses between the differentiated MST metrics and Chinese word reading measures within the DD group to determine whether neural impairments positively correlate with severity of reading difficulty. We hypothesize that children with DD will demonstrate altered spectral power and brain network topology compared to TD children, reflecting disrupted neural mechanisms. Furthermore, we expect language familiarity to modulate these neural patterns, with brain network properties correlating with reading performance, offering valuable insights into the neural basis of DD.

## Method

### Participants

In this study, we enrolled 136 participants, 85 children with DD and 51 TD children, all in the second or third grade (aged 7–11 years), to assess differences in brain spectral power and topology during resting and task states. The study was part of a larger research project approved by The Joint Chinese University of Hong Kong—New Territories East Cluster Clinical Research Ethics Committee (The Joint CUHK-NTEC CREC). All participants were native Cantonese-speaking Chinese readers recruited from primary schools and education authorities in Hong Kong. Written consent obtained from both the children and their guardians.

Children with DD had to meet the following inclusion criteria: a formal diagnosis of DD by an educational or clinical psychologist using the Hong Kong Test of Specific Learning Difficulties in Reading and Writing for Primary School Students—Third Edition [HKT-P(III)], requiring adequate IQ (85 or higher), poor literacy (− 1 SD or below), and at least one area of cognitive-linguistic deficit (− 1 SD or below); and no history of brain injury, birth complications, significant sensory impairment, or other neurological or psychological disorders (e.g., ADHD). TD children were identified based on their parents’ reports that they had no reading or writing difficulties. Detailed demographic information for the participants is provided in Table 1.

**Table 1:** Descriptive statistics and demographic information

Measurement	TD	DD	t
Male-to-female ratio	25:26	42:43	-
Age	8 (± 0.52)	8.05 (± 1.48)	−0.24
Family income^1^	3.87 (± 1.65)	3.4 (± 1.91)	1.34
Maternal education^2^	3.07 (± 1.6)	2.98 (± 1.76)	0.25
Paternal education ^3^	3.09 (± 1.67)	2.87 (± 1.96)	0.62
D-prime (Chinese)	3.87 (± 0.54)	3.47 (± 0.81)	3.16**
D-prime (Korean)	2.98 (± 0.82)	2.47 (± 0.84)	3.26**
Reading accuracy	90.23 (± 15.79)	45.2 (± 25.88)	11.46***
Reading fluency	153.28 (± 46.76)	57.67 (± 36.19)	11.68***
Chinese reaction time (ms)	678.47 (± 132.33)	764.18 (± 139.58)	−3.20**
Korean reaction time (ms)	716.03 (± 110.29)	812.70 (± 140.34)	−3.97**

^1^Monthly family income was categorized as follows: 1 for HKD 10,000 (USD 1,280) or below, 2 for HKD 10,001–20,000 (USD 1,281–2,560), 3 for HKD 20,001–30,000 (USD 2,561–3,840), 4 for HKD 30,001–40,000 (USD 3,841–5,120), 5 for HKD 40,001–50,000 (USD 5,121–6,400), and 6 for HKD 50,001 (USD 6,401) or above

^2^Maternal and paternal educational levels were coded with the following scale: 1 for middle school or below, 2 for high school, 3 for preparatory school, 4 for college, and 5 for postgraduate studies

**p <.01 (two-tailed); ***p <.001 (two-tailed)

### Behavioral Measures

Chinese word reading accuracy (Wang et al. 2021): The evaluation of Chinese word reading used a list of two-character words derived from the Hong Kong Corpus of Primary School Chinese (Leung and Lee 2002). The second character of each word progressively increased in difficulty. Children were asked to read the second character aloud and were awarded a point for each correct response. The task ended after the participant provided ten consecutive incorrect responses. The total number of correct responses was the score of Chinese word reading accuracy.

Chinese character and word reading fluency (Siu et al. 2018): Reading fluency in Chinese character and word recognition was assessed through three sub-tests. The first sub-test involved characters with pronunciations consistent with their phonetic radicals, while the second sub-test dealt with characters with pronunciations inconsistent with their phonetic radicals. The third sub-test required reading two-character words. In each sub-test, children were tasked with reading correctly as many items as possible within one minute. The sum of the three sub-test scores was the score of Chinese character and word reading fluency.

### Experimental Task

The eyes-open and eyes-closed resting-state paradigms: During the resting-state session, children were directed to complete two resting-state paradigms, with their EEG activity being recorded throughout: an eyes-open condition and an eyes-closed condition. Initially, children were guided to sit comfortably and relax. In the eyes-closed condition, they were instructed to look straight ahead, close their eyes, and remain motionless for three minutes. Conversely, for the eyes-open condition, children were asked to fixate on a cross displayed on a computer screen for three minutes without engaging in any specific task.

**Fig. 1:**
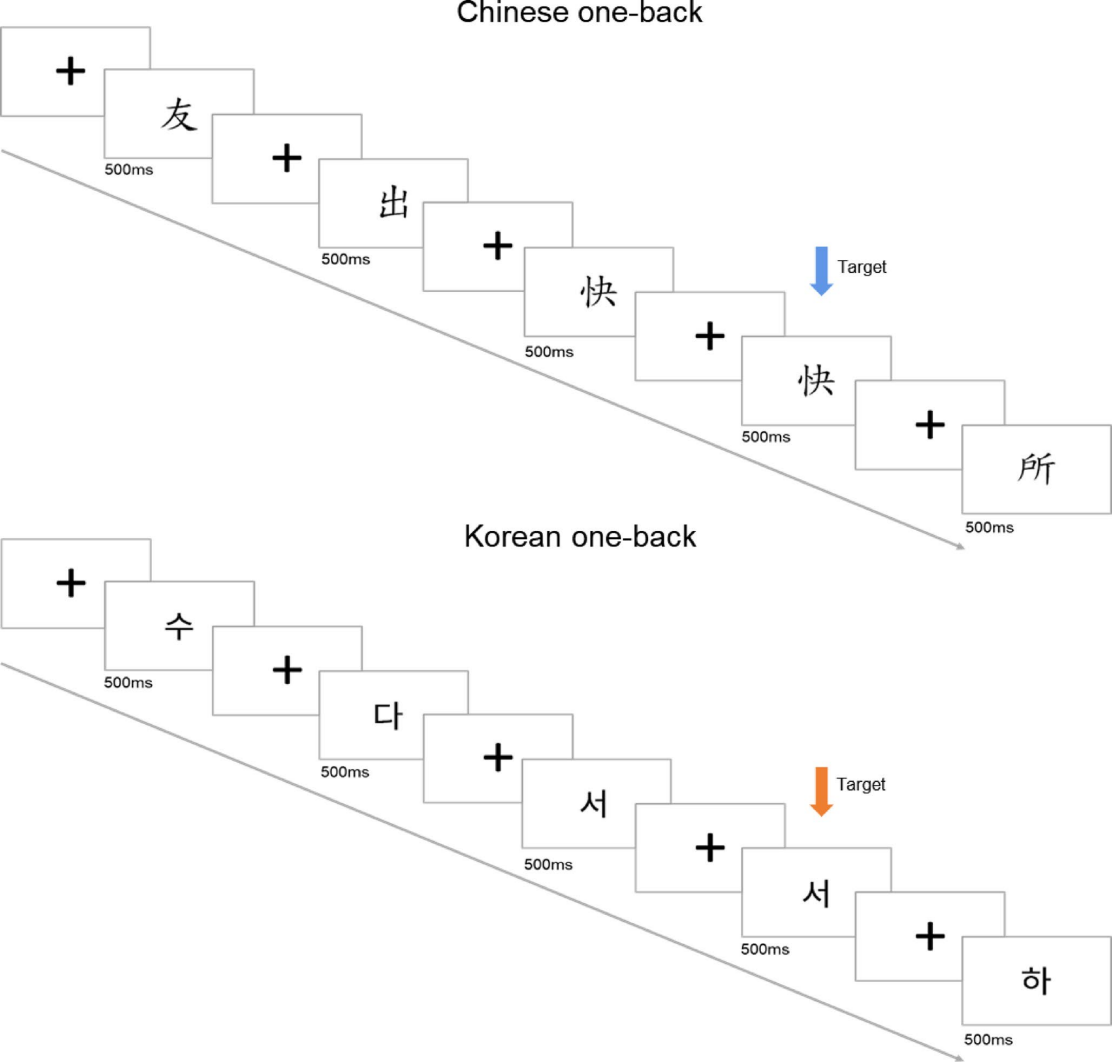
The Chinese and Korean one-back trial

Chinese-Korean character one-back task: The one-back task, adapted from Maurer et al. (2007), is known to be sensitive to reading expertise in Chinese. In this paradigm, participants were shown sequences of monosyllabic Chinese words and unfamiliar Korean characters and instructed to press the “1” key if the presented character was identical to the one shown in the preceding trial (Maurer et al. 2024; Wang and Maurer 2020; see Fig. 1). Characters from the two languages were matched in stroke number and size. Participants first completed a practice session of 10 trials.

The main experiment included four blocks (two in Chinese and two in Korean) of 46 trials, each lasting about two minutes. Trials began with a prefixation period of 250 to 750 milliseconds, followed by a character presentation for 500 milliseconds and a fixation cross for 1 s between trials. Each block contained six target characters and forty non-target characters. The order of Chinese and Korean blocks was counterbalanced between participants to eliminate order effects.

To measure discriminative performance, we utilized d-prime. We calculated the rates of correct identification of targets (hits) and incorrect identification of non-targets as targets (false alarms) and then adjusted these rates to avoid skewed results using the formula d-prime = Z(hit rate) - Z(false alarm rate). We also adjusted for perfect scores following the methods outlined in Haatveit et al. (2010). Participants with d-prime scores below 1 in either Chinese or Korean condition (i.e., 9 with DD and 1 TD) were excluded.

### EEG Data Acquisition and Analysis

#### EEG Recording and Preprocessing

EEG data were recorded in resting states and during a Chinese-Korean character one-back task using a 128-channel EGI system (Electrical Geodesics, Inc.) at a sampling rate of 500 Hz. The recordings were referenced to Cz with an online filter range of 0.1–100 Hz, and impedances were maintained below 50 kΩ. Pre-processing was conducted using BrainVision Analyzer software (Brain Products GmbH). The raw EEG data were filtered offline with a 0.3–70 Hz bandpass filter and a 50 Hz notch filter to remove power line noise. Channels that consistently exhibited poor signal quality throughout the task, such as those showing unusually high-amplitude oscillatory noise or sawtooth-like waveform distortions, were identified through visual inspection and excluded from further analysis. On average, 1.49 ± 1.79 and 1.08 ± 1.01 channels were excluded in the eye-closed condition for the DD and TD groups, respectively, and 1.48 ± 1.64 and 0.83 ± 0.97 channels were excluded in the eye-open condition. For the one-back task, 2.79 ± 2.57 and 1.78 ± 2.00 channels were excluded in the DD and TD groups. The remaining data were subjected to independent component analysis (ICA) to correct eye movement artifacts. ICA was performed using the Extended Infomax algorithm, with all channels enabled and continuous data per subject. Artifactual components were identified by visual inspection of their time series, scalp topographies, and spectral profiles. On average, 4.72 ± 2.70 and 5.61 ± 2.80 components were removed in the DD and TD groups, respectively, for the resting-state eyes-closed condition; 2.54 ± 1.82 and 3.43 ± 2.45 components were removed for the eyes-open condition; 1.93 ± 0.82 and 1.80 ± 0.89 components were removed for the one-back task. Subsequently, the excluded channels were reconstructed using spline interpolation, and all channels were re-referenced to an average reference.

For the resting-state analysis, EEG data were segmented into 2-second epochs, whereas for the task-related analysis, 2-second epochs were extracted from 150 milliseconds before to 1850 milliseconds after each character onset, and only trials without a behavioral response were included in the following analysis. To refine computations and minimize volume conduction from nearby channels, 32 channels matching the 10–20 system were extracted from the initial 128-channel setup, following the transformation standards set by Luu & Ferree (2005), including channels AF3, AF4, FC1, FC2, FC5, FC6, FP1, FP2, F3, F4, F7, F8, FZ, T7, T8, C3, C4, CP1, CP2, CP5, CP6, CZ, P3, P4, P7, P8, PO3, PO4, PZ, O1, O2, and OZ. Artifact rejection criteria excluded segments exceeding ± 100 µV, and participants with fewer than 20 clean segments were excluded, resulting in the removal of 34 participants for the eyes-closed condition, 42 for the eyes-open condition, and 14 for the task-related data. The final sample included 102 participants (40 TD children, 62 children with DD) for the eyes-closed condition; 94 participants (33 TD, 60 DD) for the eyes-open condition; and 115 participants (48 TD, 66 DD) for the Chinese-Korean one-back task, following the exclusion of 10 participants due to poor task performance. Detailed demographic information of each condition is provided in Supplementary Material 1. After exclusion, 6,270 segments (M(± SD) = 61.47 ± 21.26) and 5,284 segments (M(± SD) = 56.82 ± 19.31) were retained for the eyes-closed and eyes-open conditions, respectively. For the one-back task, 6,120 Chinese (M(± SD) = 53.68 ± 13.27) and 6,383 Korean trials (M(± SD) = 55.99 ± 14.09) were retained.

Portions of the eyes-closed resting-state EEG data were used to determine the individual alpha frequency, which defined personalized frequency bands for analyzing EEG power during the working memory task to compare children with DD and TD children (Wang et al. 2022).

#### Spectral Power

We performed an in-depth spectral power analysis in resting and task states (i.e., eyes-closed, eyes-open, Chinese one-back, and Korean one-back) to investigate the differences in EEG activity between children with DD and TD children across the four experimental conditions: resting-state eyes-closed, resting-state eyes-open, one-back Chinese, and one-back Korean conditions. For this analysis, we utilized the Fast Fourier Transform (FFT) technique, as described by Brigham (1988), to calculate the spectral power for each EEG channel and segment, achieving a frequency resolution of 0.5 Hz. Each 2‑s segment (*N* = 1000 samples at 500 Hz) was tapered with a Hann window of length N and then submitted to a single N‑point FFT without overlap. EEG signals were filtered using bandpass filters to isolate frequency bands, and a windowing function was applied to minimize spectral leakage before FFT computation. The absolute power for the alpha (8–12 Hz) and beta (12–30 Hz) bands was calculated as the sum of the squared amplitudes of the FFT results. We focused on alpha given its well-established links to working memory during nback tasks and to attentional control during resting-state (Chikhi et al. 2022; Wang et al. 2022), and on beta because of its association with executive functions and visuomotor integration—domains often impaired in DD (Basharpoor et al. 2022). These spectral power values were then averaged across epochs for different scalp areas of interest, including central (C3, C4, CP1, CP2, CP5, CP6, CZ), frontal (AF3, AF4, F3, F4, F7, F8, FC1, FC2, FC5, FC6, FP1, FP2, FZ), temporal (T7, T8), parietal (P3, P4, P7, P8, PO3, PO4, PZ), and occipital (O1, O2, OZ) scalp areas. Delta (0.5–4 Hz) and theta (4–8 Hz) power were also computed for exploratory purposes.

#### Functional Connectivity

Before constructing MST graphs to quantify brain topological structures, it is essential to compute measures of FC. In this analysis, we employed the phase-lag index (PLI) to quantify FC across all pairwise combinations of the 32 channels. The PLI is a statistical measure designed to capture phase synchronization by assessing the asymmetry in the distribution of instantaneous phase differences between two signals (Stam et al. 2007). A uniform distribution of phase differences indicates an absence of phase synchronization between EEG signals, whereas any deviation from uniformity (i.e., a nonuniform or asymmetric distribution) denotes phase locking and interdependence of the underlying sources. The PLI is less susceptible to confounding influences such as volume conduction and electrode montage. The PLI is defined as follows:

$$\:PLI=\left|\text{s}ign\left[{\Delta\:}{\upphi\:}\left({t}_{k\:}\right)\right]\right|$$

The PLI ranges from 0 to 1, where a value of 0 indicates that the phase difference between the two signals is uniformly distributed around zero (i.e., no asymmetry). In contrast, a value of 1 indicates that the phase difference is either always positive or consistently negative (i.e., complete asymmetry).

In our study, the PLI was computed to assess FC within the alpha and beta bands across resting and task states within the MATLAB R2018b environment (MathWorks Inc., Natick, MA, USA), utilizing the HERMES brain connectivity toolbox (Niso et al., 2013), which is available at https://hermes.med.ucm.es. We also derived PLI FC matrices for the delta and theta bands for an exploratory analysis. A 32 × 32 connectivity matrix was generated for each segment, condition, and frequency band. MST graphs were then constructed based on the PLI matrix of each segment to characterize the underlying brain topology.

#### Network Topology

**Fig. 2:**
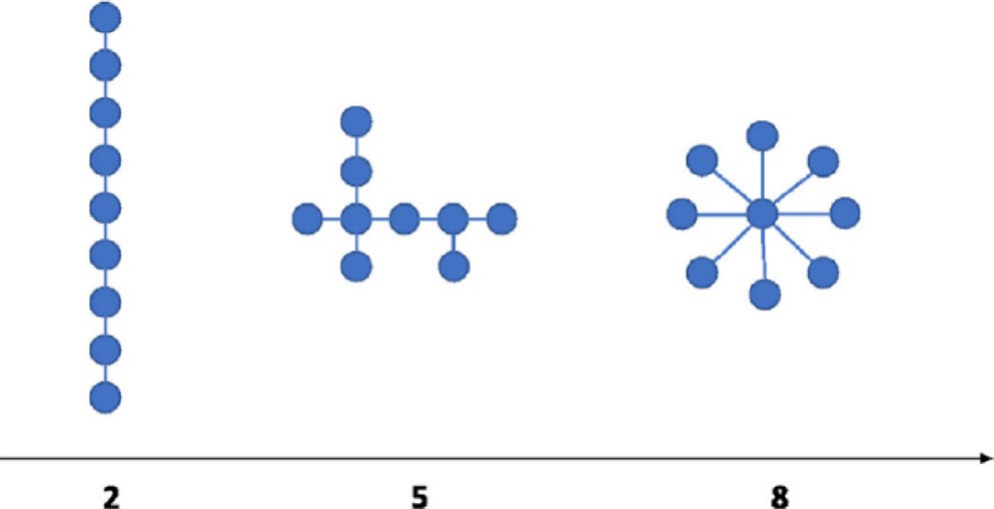
Tree topologies and MST examples in brain networks. Illustrative progression of tree structures ranging from a path-like topology (left) to a star-like topology (right). Each example contains nine nodes (depicted as circles) and eight edges (illustrated as lines). This series demonstrates an increase in the number of leaf nodes, where the path-like tree exemplifies maximal segregation, and the star-like tree represents maximal integration within a network. Adapted from Fraga-González et al. (2016)

To quantify the topology of brain networks, we constructed an MST for each segment-derived PLI matrix. This segment-wise approach follows standard practice in the MST literature (González et al. 2016; Xue et al. 2020; Zhang et al. 2022) and preserves the natural, moment-to-moment fluctuations in network topology. In principle, MST graphs were expected to display an intermediate topology between two extreme cases: a path-like topology (corresponding to maximal segregation) consisting of a series of successively connected nodes and a star-like tree (corresponding to maximal integration) characterized by a central node to which all other nodes are connected with only one edge (see Fig. 2). An MST with N nodes contains N-1 edges, and the weight of each edge is defined as 1 - PLI, and the edges are then sorted in ascending order. This transformation was necessary because MST construction seeks to minimize the total cost required to connect all nodes. By converting PLI values into a distance-like metric, stronger functional connections yield lower weights, enabling the MST algorithm to prioritize these during optimization. The Kruskal algorithm (Kruskal 1956) was used to add the edge with the lowest weight to the tree or skip it to avoid forming a loop until all nodes were connected in a loopless network (Stam et al. 2014).

To evaluate the topological properties of the MST network in our study, we also computed various topology metrics, including Degree, Kappa, Betweenness Centrality (BC), Diameter, Eccentricity (Ecc), Leaf Fraction (LF), and Tree Hierarchy (Th). These metrics were widely used in previous brain network topology studies of dyslexia (Fraga González et al. 2018; Xue et al. 2020). Table 2 offers detailed descriptions of these metrics.

**Table 2:** MST metrics summary

MST Metrics	Description
Degree	The number of neighbors for a given node
Kappa	The measure of the broadness of the degree distribution
BC	Fractions of all shortest paths that pass through a given node
Diameter	The largest distance between any two nodes
Ecc	The largest distance between a given node and any other node
LF	Fraction of nodes with a degree of 1
Th	A hierarchical metric quantifying the trade-off between large-scale integration in the MST and the overload of central nodes

Abbreviations: BC = Betweenness Centrality, Ecc = Eccentricity, LF = Leaf Fraction, Th = Tree Hierarchy

### Statistical Analysis

We conducted independent sample t-tests to compare the behavioral performance between children with DD and TD children, including Chinese word reading accuracy and fluency, d-prime scores and reaction times for Chinese and Korean conditions in the one-back task.

For the EEG data, to address variability in segment counts across participants, we standardized our analysis by randomly selecting 20 segments from each participant for each condition, repeating this process 1000 times (Zhang et al. 2022). We then conducted independent sample t-tests and repeated-measures ANOVAs (RM ANOVAs) on the grand average of these 1000 randomly selected epoch sets for each condition. Our EEG data analysis primarily focused on the alpha and beta bands. We used independent sample t-tests to investigate group differences between children with DD and TD children concerning spectral power and MST metrics in the resting state. For the Chinese–Korean one-back task, we ran 2 × 2 RM ANOVAs (Group: DD vs. TD; Condition: Chinese vs. Korean) separately for alpha and beta spectral power at each scalp area and for each MST metric within those bands.

Shapiro-Wilk tests indicated a non-normal distribution for most spectral power and MST metrics. To achieve normality, we applied a natural log transformation to the data, consistent with methodologies utilized in preceding studies (Fraga González et al. 2016; Xue et al. 2020; Zhang et al. 2022). Additionally, to control the risk of Type I errors from multiple comparisons, we used the Benjamini–Hochberg false discovery rate (FDR) procedure (q = 0.05, two—sided; Benjamini & Hochberg, 1995). In our spectral‑power analyses, all p‑values from the five scalp areas within each frequency band were adjusted simultaneously. For the repeated‑measures ANOVAs, we applied FDR correction separately to the five regional p‑values for each main effect (group, time) and for the group × time interaction. The same FDR approach was applied to the MST metrics within each band, maintaining sensitivity to true effects while controlling the expected proportion of false discoveries.

Given our particular interest in how differentiated MST metrics relate to Chinese word reading abilities in the DD group—which may reflect underlying deficiencies in network topology related to behavioral outcomes—we performed permutation-based correlation analyses. FDR correction was also implemented here to improve the statistical robustness of the correlation results.

## Results

### Behavioral Results

Independent‑samples t‑tests revealed strong group differences in reading accuracy and fluency, with children with DD performing markedly worse than their TD peers. Likewise, in the one‑back task, the DD group exhibited significantly lower D-prime scores and longer reaction times across both Chinese and Korean conditions (all *p* <.01), reflecting poorer task performance and slower processing speed relative to TD children. Detailed behavioral results are presented in Table 1.

### Spectral Power Results

#### Resting-State Spectral Power

**Fig. 3:**
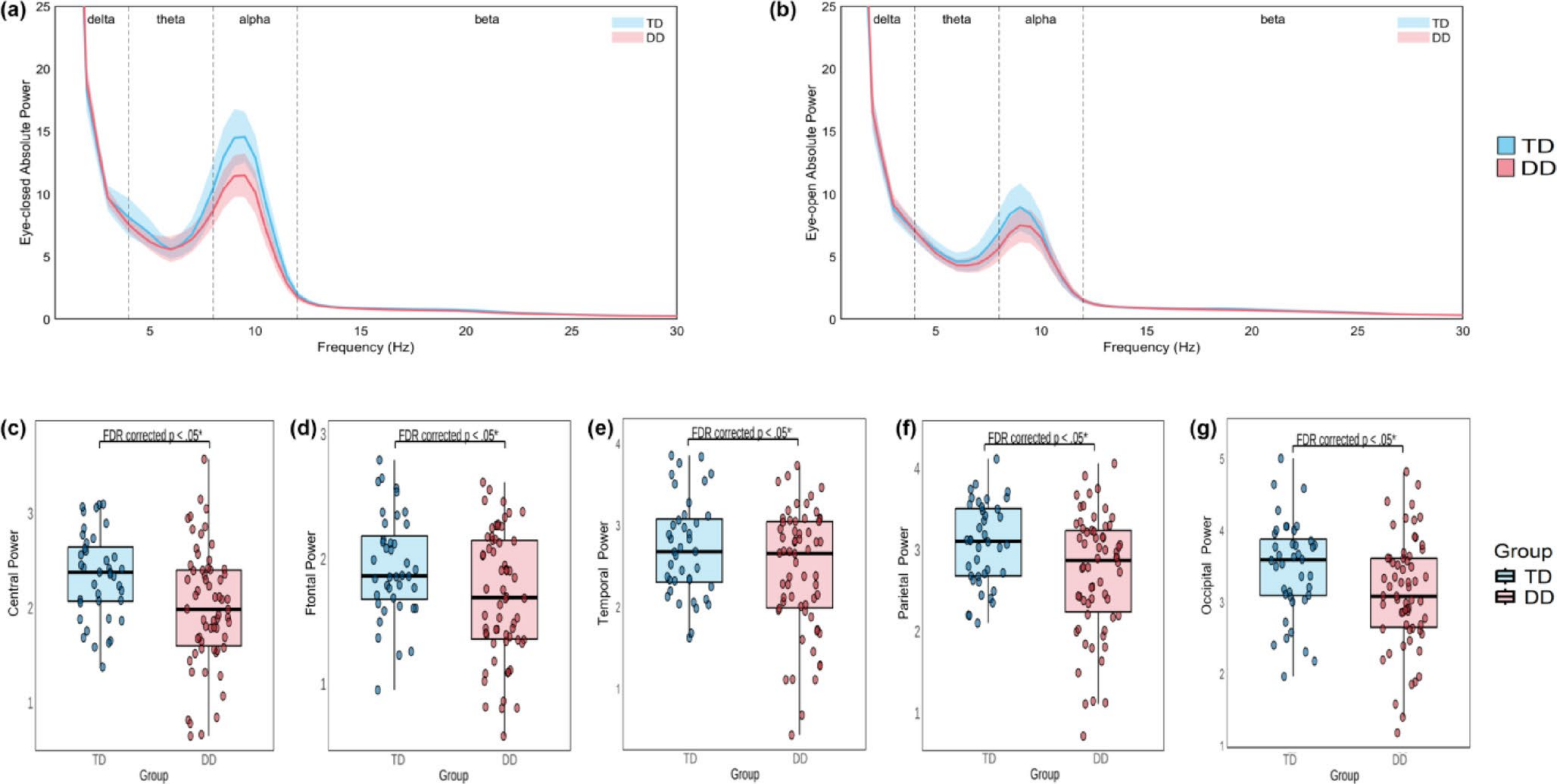
Comparison of Resting-State Spectral Power. (**a**) Average spectral power across all channels in the eyes-closed condition. (**b**) Average spectral power across all channels in the eyes-open condition. Shaded areas represent the 95% confidence interval. Comparison of log-transformed absolute power values across the central (**c**), frontal (**d**), temporal (**e**), parietal (**f**) and occipital (**g**) scalp areas in the eyes-closed alpha band, with significant differences indicated after FDR correction. Abbreviations: TD = typically developing, DD = developmental dyslexia. Significance markers: *p <.05

Significant group differences were primarily observed in the eyes-closed alpha band across the central (*t*_(100)_ = 3.136, FDR-corrected *p* =.011; Fig. 3c), frontal (*t*_(100)_ = 2.526, FDR-corrected *p* =.022; Fig. 3d), temporal (*t*_(100)_ = 2.099, FDR-corrected *p* =.038; Fig. 3e), parietal (*t*_(100)_ = 2.924, FDR-corrected *p* =.011; Fig. 3f), and occipital scalp areas (*t*_(100)_ = 2.412, FDR-corrected *p* =.022; Fig. 3g), as shown in Table 3. These effects remained significant after FDR correction, with TD children showing higher alpha power than those with DD. For clarity, only eyes-closed alpha band results are presented in Table 3. No significant group differences were found in the eyes-closed beta band; detailed results are reported in Supplementary Material 2.

**Table 3:** Group comparison of the resting-state spectral power in various scalp areas

Frequency band	Scalp area	TD		DD		Stats	
		M	SD	M	SD	t	p
Eyes-closed Alpha	Central	2.343	0.459	2.004	0.630	3.136	0.002**
	Frontal	1.939	0.413	1.708	0.506	2.526	0.013*
	Temporal	2.717	0.599	2.435	0.749	2.099	0.038*
	Parietal	3.070	0.511	2.707	0.740	2.924	0.004**
	Occipital	3.469	0.681	3.119	0.768	2.412	0.018*

Bold text indicates significant effects. Significance markers: * *p* <.05, ** *p* <.01, *** *p* <.001

In eyes-open condition, the TD group exhibited slightly higher alpha power than the DD group over the central (*t*_(91)_ = 1.746, *p* =.085) and frontal scalp areas (*t*_(91)_ = 1.731, *p* =.088), and lower beta power over the temporal area (*t*_(91)_ = −1.720, *p* =.090). This trend is also reflected in the average spectral power plots (Fig. 3a–b), where between-group differences are more pronounced in the eyes-closed condition—particularly in the alpha band—compared to the eyes-open condition. No other significant effects were observed across scalp areas in the alpha or beta bands. Full results for the eyes-open condition are available in Supplementary Material 2.

#### Task-State Spectral Power

**Fig. 4:**
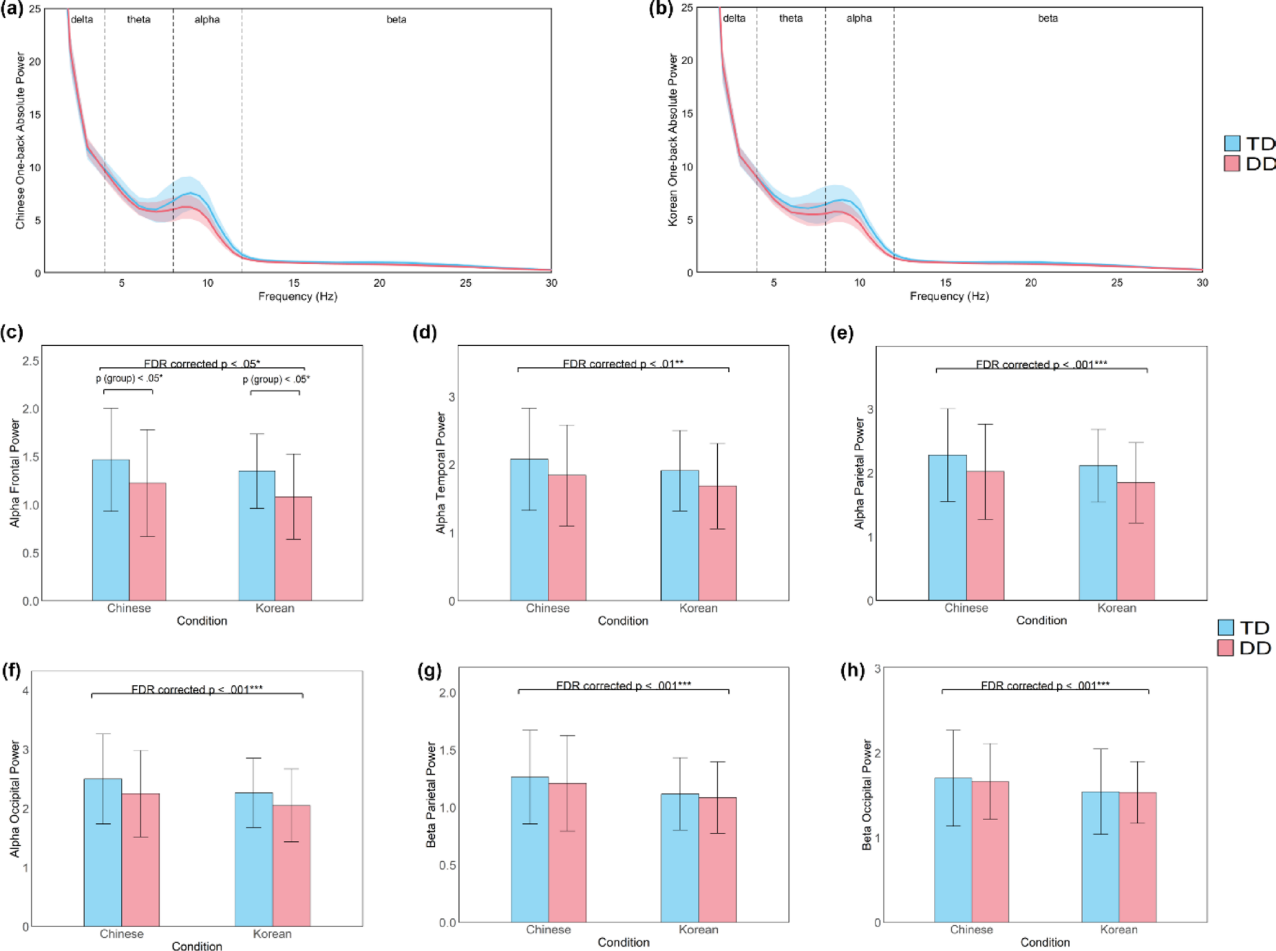
EEG spectral power differences between DD and TD children in the Chinese and Korean One-Back Task. Group averages for the alpha and beta band spectral powers: (**a**) Average spectral power across all channels in the Chinese one-back condition. (**b**) Average spectral power across all channels in Korean one-back condition. Shaded areas represent the 95% confidence interval. Group averages for the spectral power in the alpha band in the frontal (**c**), temporal (**d**), parietal (**e**), and occipital (**f**) scalp areas, and beta band in the parietal (**g**) and occipital (**h**) in the Chinese and Korean conditions of the one-back task. Abbreviations: TD = typically developing, DD = developmental dyslexia. Significance markers: *p <.05, **p <.01, ***p <.001

The RM ANOVAs revealed statistically significant differences in EEG spectral power between groups and conditions during the one-back task (see Table 4; Fig. 4). In the alpha band, significant main effects of group were observed in the central (*F*_(1,113)_ = 4.347, *p* =.039, *η*^*2*^ = 0.037) and frontal (*F*_(1,113)_ = 6.506, *p* =.012, *η*^*2*^ = 0.054) scalp areas, with the TD group showing higher alpha power compared to the DD group. However, these effects did not survive FDR correction. Marginally significant group effects were found over the temporal, parietal, and occipital scalp areas. For the main effect of condition, significant differences were identified after FDR correction in the frontal (*F*_(1,113)_ = 5.242, FDR-corrected *p* =.30, *η*^*2*^ = 0.044; Fig. 4c), temporal (*F*_(1,113)_ = 15.110, FDR-corrected *p* <.001, *η*^*2*^ = 0.118; Fig. 4d), parietal (*F*_(1,113)_ = 20.299, FDR-corrected *p* <.001, *η*^*2*^ = 0.152; Fig. 4e), and occipital (*F*_(1,113)_ = 51.263, FDR-corrected *p* <.001, *η*^*2*^ = 0.312; Fig. 4f) scalp areas, with higher alpha power in the Chinese condition compared to the Korean condition. No significant interactions were observed in the alpha band.

**Table 4:** Group and condition effects on the alpha and beta power in the one-back task

Frequency band	Scalp area	Group			Condition			Condition × Group		
		F_(1, 113)_	p	η^​2^	F_(1, 113)_	p	η​^2^	F_(1, 113)_	p	η​^2^
One-back Alpha	Central	**4.347**	0.039*	.**037**	0.110	0.741	0.001	1.697	0.195	0.015
	Frontal	**6.506**	0.012*	**0.054**	**5.242**	0.024*	**0.044**	0.709	0.401	0.006
	Temporal	2.872	0.093	0.025	**15.110**	< 0.001***	**0.118**	0.087	0.768	0.001
	Parietal	3.813	0.053	0.033	**20.299**	< 0.001***	**0.152**	0.049	0.826	< 0.001
	Occipital	2.820	0.096	0.024	**51.263**	< 0.001***	**0.312**	0.989	0.322	0.009
One-back Beta	Central	0.514	0.475	0.005	3.430	0.067	0.029	1.166	0.283	0.010
	Frontal	3.204	0.076^+^	0.028	2.167	0.144	0.019	1.648	0.202	0.014
	Temporal	0.920	0.340	0.008	0.015	0.904	< 0.001	0.092	0.762	0.001
	Parietal	0.301	0.584	0.003	**22.605**	< 0.001***	**0.167**	2.378	0.126	0.021
	Occipital	0.060	0.807	0.001	**13.741**	< 0.001***	**0.108**	1.385	0.242	0.012

Bold text indicates significant effects. Significance markers: * *p* <.05, ** *p* <.01, *** *p* <.001

In the beta band, a marginally significant main effect of group was observed over the frontal scalp area. For the main effect of condition, significant effects were found after FDR correction in the parietal (*F*_(1,113)_ = 22.605, FDR-corrected *p* <.001, *η*^*2*^ = 0.167; Fig. 4g) and occipital (*F*_(1,113)_ = 13.741, FDR-corrected *p* <.001, *η*^*2*^ = 0.108; Fig. 4h) scalp areas, demonstrating higher beta power in the Chinese condition compared to the Korean condition. A marginally significant main effect of condition was observed in the central scalp area. No significant interactions were found between the group and the condition in the beta band.

### Brain Network Topology

#### Resting-State Network Topology

**Fig. 5:**
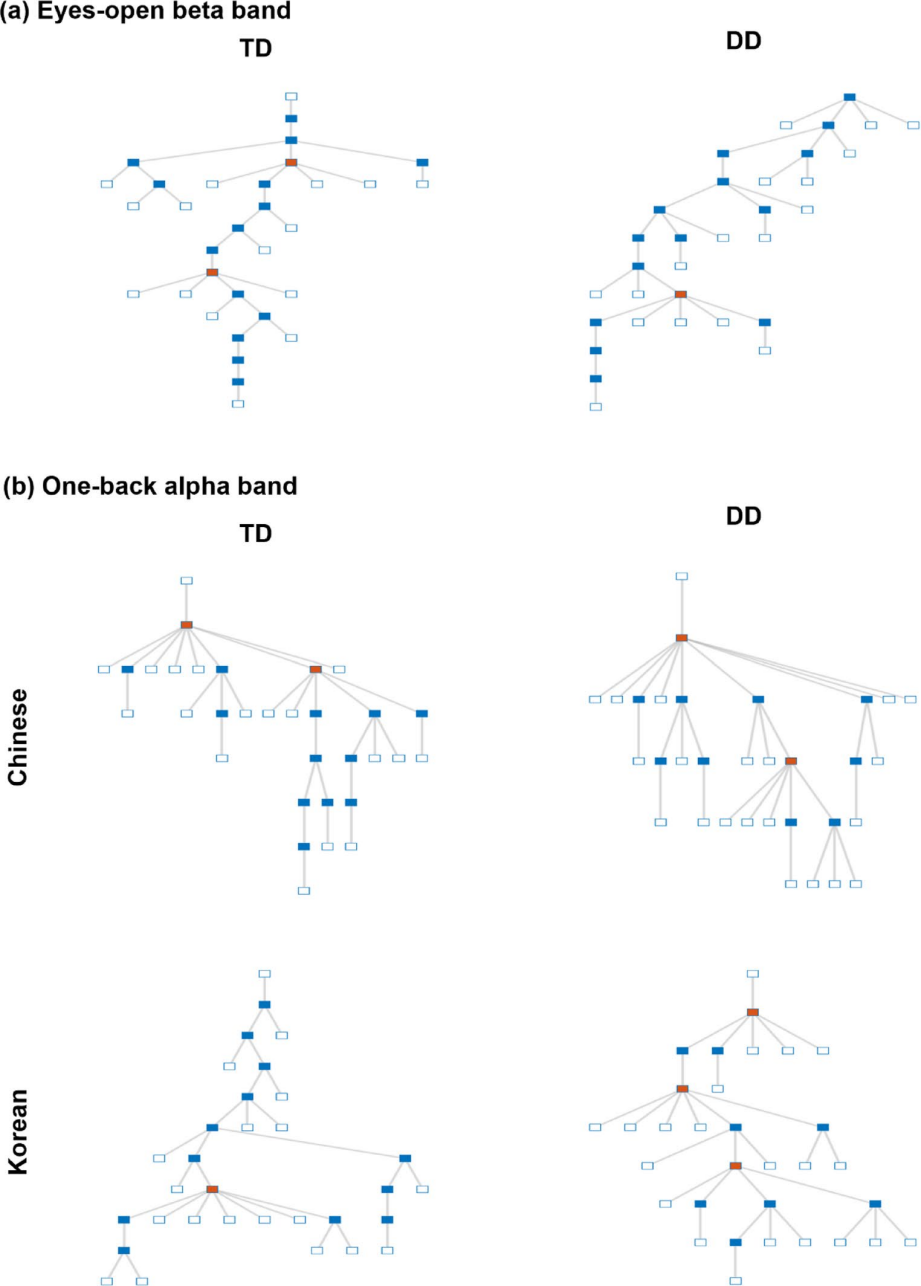
MST plots for eyes‑open beta and one‑back alpha bands. An illustration of MSTs was created using MATLAB’s treeplot function on group‑average PLI matrices for the eyes‑open beta (**a**) and one‑back alpha (**b**) bands. Abbreviations: TD = typically developing; DD = developmental dyslexia

Resting-state topology analysis in the eyes-open beta band revealed group differences in degree, Kappa, LF, and BC (see Table 5), with children with DD showing lower degree (*t*_(91)_ = 2.04, *p* =.045), Kappa (*t*_(91)_ = 2.38, *p* =.020), LF (*t*_(91)_ = 2.28, *p* =.026), and higher BC (*t*_(91)_ = − 2.03, *p* =.047) compared to the TD children. However, these effects did not survive FDR correction. No significant differences were observed in the eyes‑open alpha band or the eyes‑closed alpha or beta bands (see Supplementary Material 3). Figure 5(a) illustrates the eyes‑open beta MST; MSTs for the remaining bands are available in Supplementary Material 6.

**Table 5:** Group comparison of the beta band MST metrics during the eyes-open resting state

Frequency band	MST	TD		DD		Stats	
	Metrics	M	SD	M	SD	t	p
Eyes-open Beta	Degree	1.593	0.026	1.581	0.028	**2.040**	0.045*
	Kappa	0.174	0.042	0.152	0.044	**2.380**	0.020*
	BC	−1.919	0.020	−1.910	0.020	**−2.030**	0.047*
	Diameter	−2.163	0.057	−2.170	0.050	0.600	0.549
	Ecc	−2.461	0.056	−2.467	0.051	0.490	0.625
	LF	−0.830	0.022	−0.840	0.020	**2.280**	0.026*
	Th	2.356	0.023	2.347	0.021	1.950	0.056

Degree = Maximum Nodal Degree, BC = Betweenness Centrality, Ecc = Eccentricity, LF = Leaf Fraction, Th = Tree Hierarchy. Bold text indicates significant effects. Significance markers: * *p* <.05

#### Task-State Network Topology

**Fig. 6:**
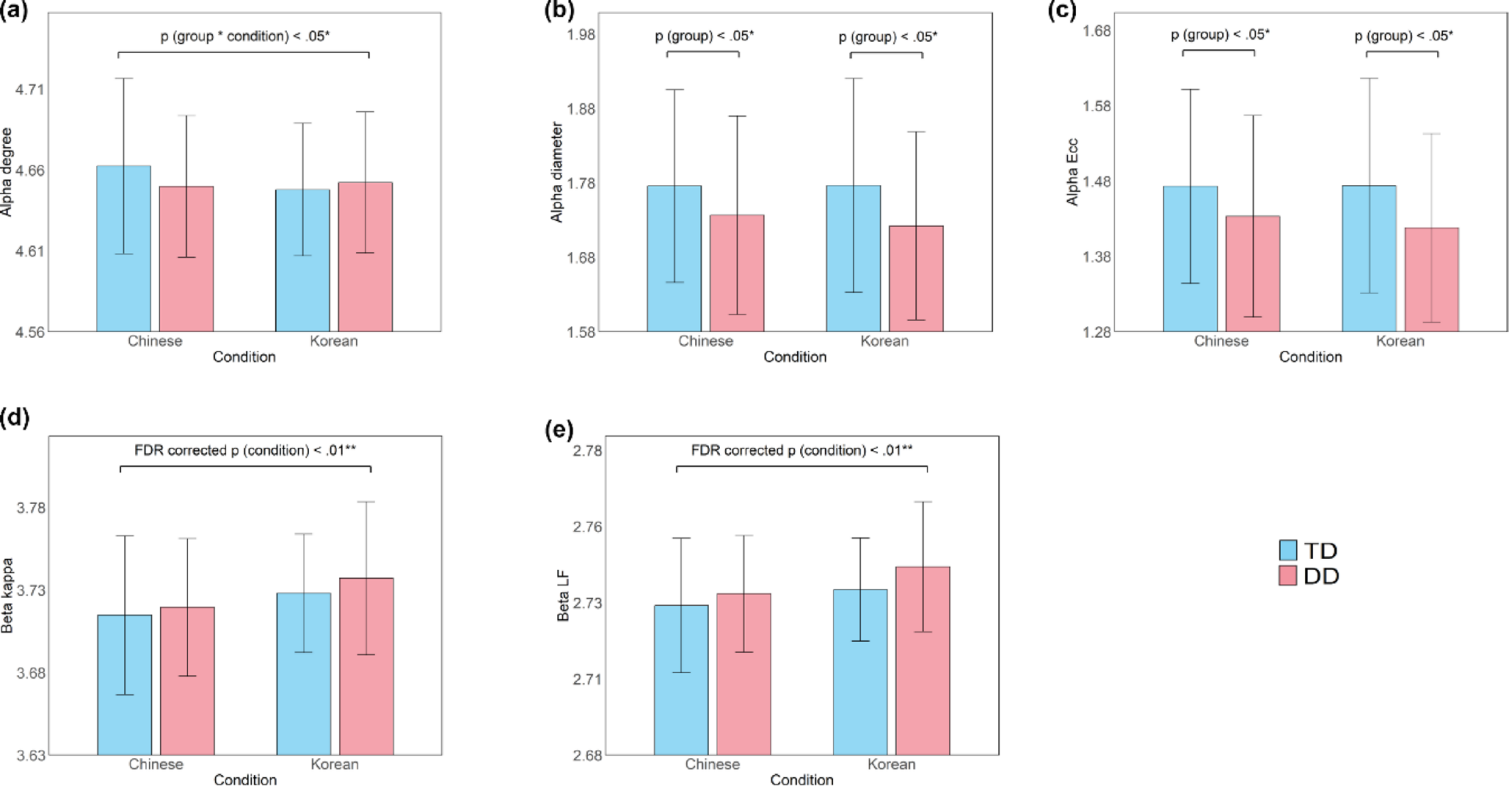
EEG MST metrics differences between DD and TD children in the Chinese and Korean One-Back Task. Group averages for the alpha and beta band MST metrics: degree (**a**), diameter (**b**), and eccentricity (**c**) of alpha band, kappa (**d**) and leaf fraction (**e**) of beta band in the Chinese and Korean conditions of the one-back task. For better visualization, a constant was added to adjust the log-transformed MST metrics with negative values. Abbreviations: Ecc = Eccentricity, LF = Leaf Fraction, MST = Minimum Spanning Tree, TD = typically developing, DD = developmental dyslexia. Significance markers: ^*^p <.05, ^**^p <.01

The analysis of network topology using RM ANOVAs uncovered significant differences between TD and DD children during the one-back task (see Table 6). The results revealed a significant main effect of the group in the diameter (*F*_(1,113)_ = 3.993, *p* =.048, *η*^*2*^ = 0.034; Fig. 6b) and eccentricity (*F*_(1,113)_ = 4.252, *p* =.036, *η*^*2*^ = 0.036; Fig. 6c) of alpha band, indicating a more integrated brain topology in the DD group than in the TD group when engaged in the one-back task. However, these effects did not survive FDR correction. Furthermore, we found a significant interaction between group and condition in the alpha band degree (*F*_(1,113)_ = 4.272, *p* =.041, *η*^*2*^ = 0.036; Fig. 6a). Post hoc analysis indicated that most pairwise contrasts were non-significant (*p* >.10); however, the comparison between the Chinese and Korean conditions yielded a marginally significant effect in the TD group (*t* = 2.326, *p* =.098). For illustration, Fig. 5b displays MST trees derived from group‑average alpha band PLI in the one‑back task; one‑back beta band MSTs are provided in Supplementary Material 6.

**Table 6:** Group and condition effects on the alpha and beta band MST metrics during the one-back task

Frequency band	MST	Group			Condition			Condition × Group		
	Metrics	F_(1, 113)_	p	η​^2^	F_(1, 113)_	p	η​^2^	F(_1, 113)_	p	η​^2^
One-back Alpha	Degree	0.310	0.579	0.003	2.202	0.141	0.019	**4.272**	0.041*	**0.036**
	Kappa	0.178	0.674	0.002	1.665	0.200	0.015	2.319	0.131	0.020
	BC	0.248	0.620	0.002	0.172	0.679	0.002	0.391	0.533	0.003
	Diameter	**3.993**	0.048*	**0.034**	0.532	0.467	0.005	0.678	0.412	0.006
	Ecc	**4.252**	0.042*	**0.036**	0.617	0.434	0.005	0.791	0.376	0.007
	LF	0.050	0.823	< 0.001	0.106	0.745	0.001	0.050	0.823	< 0.001
	Th	0.010	0.919	< 0.001	0.151	0.698	0.001	0.123	0.726	0.001
One-back Beta	Degree	0.052	0.820	< 0.001	1.751	0.188	0.015	0.004	0.951	< 0.001
	Kappa	1.174	0.281	< 0.001	**9.066**	**0.003** ^**^	**0.074**	0.176	0.676	0.002
	BC	0.605	0.438	0.005	3.220	0.075^+^	0.028	0.009	0.924	< 0.001
	Diameter	0.426	0.515	0.004	0.023	0.881	< 0.001	0.054	0.816	< 0.001
	Ecc	0.215	0.643	0.002	0.010	0.922	< 0.001	0.121	0.728	0.001
	LF	3.813	0.053^+^	0.033	**8.296**	**0.005** ^**^	**0.068**	0.563	0.455	0.005
	Th	2.279	0.134	0.020	2.358	0.127	0.020	1.308	0.255	0.011

Degree = maximum nodal degree; BC = betweenness centrality; Ecc = eccentricity; LF = leaf fraction; Th = tree hierarchy. Bold text indicates significant effects. Significance markers: * *p* <.05, ** *p* <.01, *** *p* <.001

Additionally, a significant main effect of the condition survived FDR correction in the beta band, showing lower Kappa (*F*_(1,113)_ = 9.066, FDR-corrected *p* =.017, *η*^*2*^ = 0.074; Fig. 6d) and LF (*F*_(1,113)_ = 8.296, FDR-corrected *p* =.017, *η*^*2*^ = 0.068; Fig. 6e) in the Chinese condition compared to the Korean condition (see Table 6; Fig. 5). Marginally significant main effects of the condition were also found in the BC and Th metrics.

### Exploratory Results in the Delta and Theta Bands

We conducted exploratory analyses of delta and theta band activity during the resting-state (eyes-open and eyes-closed) conditions. No significant main effects of group were observed for theta or delta spectral power or MST metrics in either condition. Full statistical details are presented in Supplementary Material 4.

Additional exploratory analyses were performed for the one-back task. Repeated-measures ANOVA revealed a significant main effect of condition in the delta band over the central (*F*_(1,113)_ = 19.187, FDR-corrected *p* <.001, *η²* = 0.145), parietal (*F*_(1,113)_ = 7.130, FDR-corrected *p* =.022, *η²* = 0.059), and occipital (*F*_(1,113)_ = 6.270, FDR-corrected *p* =.023, *η²* = 0.053) scalp areas. In the theta band, significant condition effects were found in the central (*F*_(1,113)_ = 9.697, FDR-corrected *p* =.002, *η²* = 0.145), frontal (*F*_(1,113)_ = 10.900, FDR-corrected *p* =.002, *η²* = 0.044), temporal (*F*_(1,113)_ = 38.428, FDR-corrected *p* <.001, *η²* = 0.254), parietal (*F*_(1,113)_ = 83.320, FDR-corrected *p* <.001, *η²* = 0.424), and occipital scalp areas (*F*_(1,113)_ = 65.822, *p* <.001, *η²* = 0.368), indicating higher spectral power in the Chinese condition compared to the Korean condition. No significant main effects of group or interactions were found in the spectral power results. For MST metrics, a significant main effect of condition was observed in the delta band diameter (*F*_(1,113)_ = 4.905, *p* =.029, *η²* = 0.042) and eccentricity (*F*_(1,113)_ = 4.127, *p* =.045, *η²* = 0.035), with both measures higher in the Chinese condition compared to the Korean condition. However, these effects did not survive FDR correction. No other significant group effects or interactions were observed, and no significant results were found in the theta band MST metrics. Detailed results are provided in Supplementary Material 5.

### MST Metrics and Chinese Word Reading Performance

We conducted permutation-based correlation analysis with FDR correction to investigate the relationship between differentiated MST metrics (i.e., beta band MST metrics during eyes-open resting state and alpha band metrics during the one-back task) and Chinese word reading performance within the DD group. For a comprehensive table of all computed correlations, please refer to Supplementary Materials 7 and 8.

**Fig. 7:**
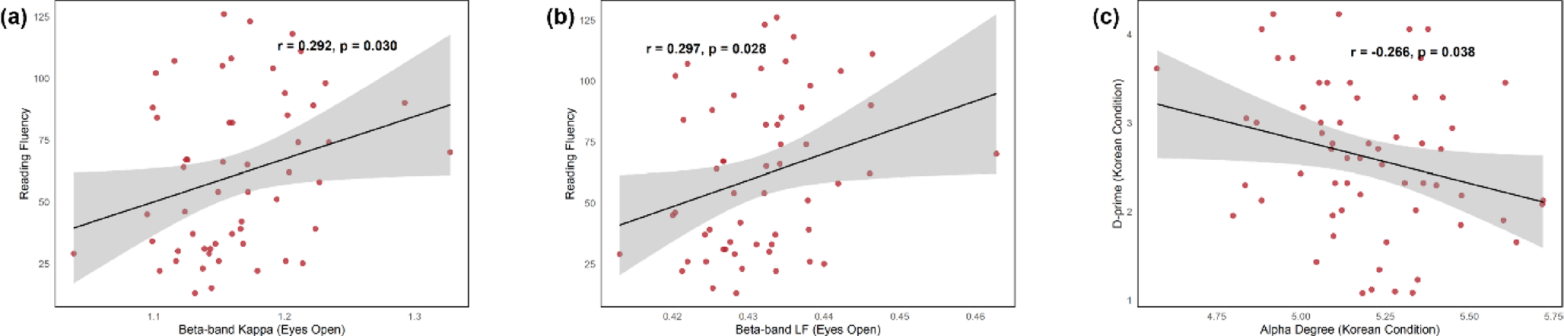
Significant correlations between MST metrics and Chinese reading performance in the group with developmental dyslexia. Correlations between MST metrics and Chinese reading performance in the group with developmental dyslexia: (**a**) eyes-open beta band Kappa metric and reading fluency, (**b**) eyes-open beta band Leaf Fraction metric and reading fluency, and (**c**) alpha degree during the one-back task and d-prime in the Korean condition. Abbreviations: LF = Leaf Fraction; MST = Minimum Spanning Tree. ^*^p <.05

The results revealed a positive correlation between the eyes-open beta band Kappa metric and reading fluency (*r* =.292, *p* =.030; Fig. 7a). Additionally, a significant positive correlation was observed between the eyes-open beta band Leaf Fraction metric and reading fluency (*r* =.297, *p* =.028; Fig. 7b). However, in the one-back task, a negative association was found between the alpha band degree metric and d-prime in the Korean condition (*r* = −.266, *p* =.038; Fig. 7c). These findings, however, did not remain significant after FDR correction.

## Discussion

The current study provides compelling evidence of neural abnormalities in Hong Kong children with DD, highlighting differences in EEG spectral power and brain network topology during both resting and task states. Key findings during the resting state indicate reduced alpha power and a less integrated beta band network. During the one-back task, spectral power analyses revealed increased alpha power and more integrated brain networks in children with DD compared to their TD peers. For familiar stimuli (Chinese characters), significant increases in alpha and beta power were observed across multiple brain areas, alongside less integrated alpha and beta networks compared to the Korean condition. Additionally, an interaction effect in the alpha band degree between group and condition suggests that the two groups exhibit different neural responses when switching between the Chinese and Korean conditions. Exploratory correlation analyses further revealed that differentiated brain metrics have potential behavioural relevance. Collectively, these findings elaborate on the neural underpinnings of DD in Chinese children during both resting and task states, suggesting that alpha band spectral power may be a stable biomarker for Chinese DD. Greater network integration could act as a compensatory mechanism in children with DD, supporting working memory and task performance. When processing unfamiliar stimuli, increased network integration may similarly serve a compensatory role.

### Reduced Alpha Power in DD Compared To TD Children Across Resting and Task States

Our analysis revealed reduced alpha power in children with DD during the eyes-closed condition, particularly in the central, frontal, temporal, parietal, and occipital scalp areas. This reduction persisted during task-state EEG, with significantly lower alpha power in the central and frontal scalp areas compared to TD children. These findings align with recent meta-analytical results, which characterize children with DD by a widespread reduction in alpha band activity across multiple scalp areas (Cainelli et al. 2023). Our results complement these findings, showing similar patterns in a Chinese cohort, thus reinforcing the generalizability of these characteristics across diverse populations.

Alpha oscillations play a key role in cognitive control by inhibiting task-irrelevant information and reducing competing neural activity (Klimesch 2012). They are crucial for reactive, modality-specific distracter suppression during working memory tasks (Zhou et al. 2023). Higher frontal alpha power has been linked to better inhibitory control (Klimesch 2012; Van der Lubbe et al. 2019). Thus, the decreased alpha activity in children with DD, particularly in the frontal area, may indicate impaired inhibitory control. In addition, Deiber et al. (2021) demonstrated that reduced alpha power reflects cortical hyperactivation, as it weakens the brain’s ability to suppress irrelevant activity, resulting in excessive and dysregulated neural responses. Similarly, our findings demonstrate that children with DD exhibit reduced alpha power during both resting and task states. This reduction may indicate a state of cortical hyperactivation, potentially contributing to cognitive impairments observed in children with DD.

Moreover, prior research has noted a positive correlation between alpha power and phonological skills in TD children (De Vos et al. 2017), which indicates that impaired synchronization within the alpha band may hinder phonological processing in children with DD. Overall, this evidence suggests that reduced alpha activity is indicative of difficulties with inhibitory control and cortical hyperactivation in children with DD. This highlights the potential of alpha band brain activity as a biomarker for underlying cognitive deficits associated with DD.

### Diminished Resting State Beta Band Network Integration in DD

Children with DD showed less integrated network topology in the eyes-open beta band, with higher degree, kappa, LF, and Th compared to TD children. This aligns with previous research on Mandarin-speaking children with DD, who also showed less integrated brain network topology in the beta band (Xue et al. 2020). Additionally, fMRI studies have shown that Chinese individuals with DD exhibit less integrated brain functional organization within structural networks than TD peers (Liu et al. 2015; Zhang et al. 2021).

Research suggests that resting beta activity is linked to the default mode of processing and ongoing cognitive activity beyond task-specific functions (Laufs et al. 2003). Studies have shown that resting-state beta activity can predict motor skill learning (Sugata et al. 2020; Wu et al. 2014). Additionally, resting-state beta plays a significant role in visual perception (Quentin et al. 2016) and can predict performance in visual tasks (Rogala et al. 2020). Impairments in these functions could contribute to difficulties in tasks requiring coordination and visual attention, which are often reported in children with DD. However, De Vos et al. (2017) reported increased beta synchronization during task performance in adolescents with DD, which was interpreted as a compensatory mechanism to enhance phonemic processing. While this task-state over-synchronization may support specific cognitive functions, resting-state beta integration likely reflects broader cognitive capacity. The less integrated beta band topology in children with DD may reflect general impairments in cognitive processing, particularly in motor coordination and visual perception.

### Enhanced Alpha Band Integration and Language Effects in One-Back Task for DD

Further, our results suggest that children with DD show decreased eccentricity and diameter during the one-back task in the alpha band. With reduced eccentricity, these children exhibited shorter distances to other nodes, indicating a more central role in the alpha band network (Stam et al. 2014; Van Dellen et al. 2014). The reduced diameter also implies a shorter node path and a more integrated network in children with DD during the one-back task (van Stam and Van Straaten 2012; Tewarie et al. 2015b). While our task-based findings point to increased alpha band integration in DD, Fraga González et al. (2018) reported decreased alpha band network integration in dyslexic adults at rest. This contrast may reflect state-dependent dynamics, where reduced integration at rest relates to lower baseline attention control, whereas increased integration during task performance may reflect compensatory recruitment to meet cognitive demands. Interestingly, previous research using MST-based brain topology on children reported higher network integration in the alpha band with increased cognitive demands during a math task (Vourkas et al. 2014). This finding may suggest a relationship between network integration and cognitive functions such as attentional and working memory processes. Enhanced alpha band integration could imply higher cognitive demand and reduced recruitment of specialized subnetworks in children with DD, potentially representing a compensatory mechanism to support tasks related to working memory. However, increased network integration does not always indicate efficient processing. It may also reflect cognitive overload or reduced network flexibility, where broader engagement compensates for inefficient routing. Similar patterns have been reported in ADHD, with elevated alpha connectivity and limited modulation with task demands (Michelini et al. 2019), and in other clinical groups showing signs of “hub overload” (Fodor et al., 2021). Thus, increased alpha band integration in DD may reflect both compensatory adaptation and underlying inefficiency.

Moreover, our study found a significant interaction in alpha band degree between group and task condition, indicating that language familiarity (Chinese vs. Korean) affects neural processing differently in children with DD compared to TD children. However, the post hoc analysis did not reveal significant differences within the specific conditions. This suggests that while there is an overall interaction effect, the subtleties of how language familiarity influences neural processing might not be strong enough to produce significant differences in specific pairwise comparisons. It could indicate variability within groups or a need for a larger sample size to detect more nuanced effects.

### Higher Alpha and Beta Power in One-Back Chinese Compared to Korean Condition

In examining the main effect of condition, we observed increased activity in the temporal, parietal, and occipital scalp areas when children were presented with Chinese stimuli compared to Korean stimuli. High alpha power is often associated with excitatory processes during cognitive events, as noted by Klimesch (2012). This is supported by findings from Bailey et al. (2018), who reported a negative correlation between parietal-temporal alpha amplitude and pseudoword reading speed. Furthermore, Jensen and Mazaheri (2010) have linked alpha power to the functional inhibition of task-irrelevant brain activity, which aids in allocating cognitive resources to task-relevant regions, thereby optimizing performance. These observations suggest that the elevated alpha power in the familiar Chinese condition might indicate more active cognitive processing with familiar Chinese words. This familiarity with Chinese likely enables children to more effectively inhibit irrelevant brain activity, requiring less cognitive engagement and enhancing processing efficiency for familiar linguistic information.

During Chinese trials, increased beta power was observed over the parietal and occipital scalp areas. Beta band oscillations represent the natural rhythms of the parietal cortices (Capilla et al. 2022; Samaha et al. 2017). Specifically, beta activity in the parietal regions is associated with various visual processes, such as visual detection, motion discrimination, and spatial attention (Stengel et al. 2021; Veniero et al. 2021). The increased beta power likely reflects enhanced visual processing when engaging with familiar Chinese words. This activation suggests automatic lexical access within the language network, facilitating faster and more efficient comprehension. By contrast, the processing of unfamiliar Korean stimuli likely requires greater cognitive effort, as automaticity is reduced or absent.

### Less Integrated Beta Band Brain Topology in Chinese Compared to Korean Condition

In the beta band, lower kappa and leaf fraction were observed in the Chinese condition compared to the Korean condition. Kappa reflects the distribution of degrees within the network, indicating diversity in node connectivity. Lower kappa values suggest less scale-free network properties, where hub nodes are less effective in facilitating efficient information flow (C. van Stam and Van Straaten 2012). Similarly, a decrease in leaf fraction indicates fewer connected hub nodes and longer path lengths, reflecting reduced efficiency in information transfer (Tewarie et al. 2015a). These findings suggest that both children with DD and TD exhibited a less integrated network topology during Chinese compared to Korean stimuli.

This indicates that processing familiar Chinese words requires lower network integration, reflecting a less demanding cognitive load compared to the unfamiliar Korean trials. The less demanding nature of the Chinese trials likely results in a less integrated brain network, as they require fewer cognitive resources for processing compared to the unfamiliar Korean stimuli.

### Correlation Between Differentiated MST Metrics and Chinese Word Reading

Lastly, we investigated the correlations between distinct resting and task-state MST metrics and Chinese word reading performance within the DD group. We found that the eyes-open resting-state beta band kappa and leaf fraction were positively associated with reading fluency. Since increased kappa and leaf fraction indicate a more integrated brain network (Zhang et al. 2022), our results suggest that a more integrated beta band topology in the resting state is linked to better reading performance. This finding aligns with previous studies showing that resting-state network modularity is related to Chinese word reading in children with typical reading development (Lui et al. 2021). The direction of the within-group correlations is consistent with the between-group comparisons, reinforcing that a more integrated resting-state brain topology benefits reading performance. However, as these correlations did not survive FDR correction, they remain exploratory.

We also observed a negative correlation between the alpha band degree metric and d-prime in the Korean condition. Increased tree degree reflects greater connectivity within the network, indicating higher network integration (Bullmore and Sporns 2009). The negative correlation suggests that higher alpha network integration may be needed to support the cognitive demands of processing unfamiliar stimuli. This supports the idea that over-integration of the alpha network could increase cognitive load, potentially reducing task efficiency.

### Limitations

Our study offers valuable insights into the abnormalities in resting-state alpha power and beta band network integration observed in children with DD, highlighting the influence of language familiarity on neural processing. However, several limitations should be addressed in future research. Although we used the PLI for functional connectivity analysis, which is robust against volume conduction effects (Stam et al. 2007), the analysis was performed at the sensor level, limiting the ability to detect cortical source-specific abnormalities. Future studies could enhance this by employing EEG source localization, allowing for more precise interpretations and cross-validation with other neuroimaging techniques, such as fMRI and MEG. Additionally, we did not directly compare state‑dependent spectral power and brain topology alterations between resting and task conditions in DD versus TD children. Future studies that explicitly test for such state‑by‑group interactions will help uncover finer‑grained neural dynamics underlying DD. Finally, because Chinese is a logographic (non-alphabetic) writing system, the extent to which these spectral and topological abnormalities generalize to alphabetic orthographies remains unclear. Comparative studies with larger, crosslinguistic cohorts that integrate both the resting-state and task-related paradigms are essential to establish the universality of these neural markers.

## Conclusion

This study highlights key neural abnormalities in Hong Kong Chinese children with DD, with a focus on spectral power and brain network topology. Children with DD showed reduced alpha power in both resting and task states—potentially indicating deficits in inhibitory control and information processing—and decreased resting-state beta band network integration, which could reflect impairments in broader cognitive processing. Conversely, the observed increase in alpha band network integration during task performance may reflect compensatory engagement of working memory networks, though this interpretation remains tentative. Language familiarity significantly modulated neural responses: higher alpha and beta power during familiar Chinese stimuli indicated more efficient and automatic processing, while decreased beta band network integration suggested reduced cognitive effort required to process familiar tasks. Additionally, the association between a more integrated resting-state beta band network and better Chinese reading performance underscores the role of brain network topology in influencing reading outcomes in children with DD.

These findings offer important insights into the neural basis of DD and suggest that spectral and topological abnormalities—particularly in the alpha band—may serve as useful neural markers for reading difficulties. This raises the potential for developing EEG-based screening tools using alpha band features. Targeted interventions, such as neurofeedback training aimed at modulating alpha and beta activity, may help improve reading and cognitive performance. Prior studies have shown that neurofeedback can enhance reading accuracy, phonological awareness, and spelling in children with dyslexia (Nazari et al. 2012; Breteler et al. 2010), offering a promising complement to traditional literacy interventions by addressing underlying neural inefficiencies. Moreover, the differential neural responses to familiar versus unfamiliar stimuli emphasize the need for language-specific intervention strategies, contributing to a better understanding of the interplay between brain function and linguistic context.

## Electronic Supplementary Material

Below is the link to the electronic supplementary material.

Supplementary Material 1

## Data Availability

The datasets generated and analyzed during the current study are available from the corresponding author upon reasonable request.
