# Supplementary material for: Spectral and Topological Abnormalities of Resting and Task State EEG in Chinese Children with Developmental Dyslexia
Source: Brain Topogr. 2025 Jun 10;38(4):50. doi: 10.1007/s10548-025-01123-0 (PMC12152076; doi:10.1007/s10548-025-01123-0)
Supplement: Supplementary file 1 — Supplementary Material 1 [file 10548_2025_1123_MOESM1_ESM.docx]

**Supplementary material**

**Supplementary Material 1**

**Demographic details in different conditions.**

| **Measurement** | **TD** | **DD** | **t-value** |
| --- | --- | --- | --- |
| Eye-closed | | | |
| Male-to-female ratio | 21:19 | 28:34 | - |
| Age | 8.03 (± 0.56) | 7.84 (± 0.72) | 1.38 |
| Family income^1^ | 3.83 (± 1.66) | 3.61 (± 1.74) | 0.61 |
| Maternal education^2^ | 3 (± 1.59) | 3.12 (± 1.62) | -0.36 |
| Paternal education ^3^ | 3.29 (± 1.71) | 2.81 (± 1.81) | 1.27 |
| D-prime (c) | 3.82 (± 0.6) | 3.35 (± 0.88) | 3.21** |
| D-prime (k) | 3 (± 0.88) | 2.21 (± 1.04) | 4.08** |
| Reading accuracy | 89.82 (± 15.76) | 45.84 (± 24.58) | 10.91** |
| Reading fluency | 154.41 (± 46.69) | 58.73 (± 35.83) | 10.88** |
| Eye-open | | | |
| Male-to-female ratio | 15:18 | 33:27 | - |
| Age | 8.06 (± 0.56) | 7.91 (± 1.54) | 0.66 |
| Family income^1^ | 3.78 (± 1.56) | 3.48 (± 1.77) | 0.82 |
| Maternal education^2^ | 3.16 (± 1.57) | 3.26 (± 1.7) | -0.3 |
| Paternal education ^3^ | 3.39 (± 1.67) | 2.88 (± 1.74) | 1.35 |
| D-prime (c) | 3.95 (± 0.51) | 3.38 (± 0.92) | 3.88** |
| D-prime (k) | 3.1 (± 0.76) | 2.26 (± 1.02) | 4.49** |
| Reading accuracy | 92.44 (± 14.38) | 46.37 (± 25.89) | 10.97** |
| Reading fluency | 160.59 (± 43.18) | 59.6 (± 38.3) | 11.05** |
| Chinese-Korean One-back | | | |
| Male-to-female ratio | 24:24 | 33: 33 | - |
| Age | 8 (± 0.52) | 8.05 (± 1.48) | -0.24 |
| Family income^1^ | 3.87 (± 1.65) | 3.4 (± 1.91) | 1.34 |
| Maternal education^2^ | 3.07 (± 1.6) | 2.98 (± 1.76) | 0.25 |
| Paternal education ^3^ | 3.09 (± 1.67) | 2.87 (± 1.96) | 0.62 |
| D-prime (c) | 3.87 (± 0.54) | 3.47 (± 0.81) | 3.16** |
| D-prime (k) | 2.98 (± 0.82) | 2.47 (± 0.84) | 3.26** |
| Reading accuracy | 90.23 (± 15.79) | 45.2 (± 25.88) | 11.46** |
| Reading fluency | 153.28 (± 46.76) | 57.67 (± 36.19) | 11.68** |

**Notes:** ^1^Monthly family income was categorized as follows: 1 for HKD 10,000 (USD 1,280) or below, 2 for HKD 10,001–20,000 (USD 1,281–2,560), 3 for HKD 20,001–30,000 (USD 2,561–3,840), 4 for HKD 30,001–40,000 (USD 3,841–5,120), 5 for HKD 40,001–50,000 (USD 5,121–6,400), and 6 for HKD 50,001 (USD 6,401) or above.

^2^Maternal and paternal educational levels were coded with the following scale: 1 for middle school or below, 2 for high school, 3 for preparatory school, 4 for college, and 5 for postgraduate studies.

^**^Significant at the .01 level (2-tailed).

**Supplementary Material 2**

**Group comparison of the resting-state spectral power in Eyes-closed Beta, Eyes-open Alpha and Eyes-open Beta bands.**

|  |  | TD |  | DD |  | Stats | |
| --- | --- | --- | --- | --- | --- | --- | --- |
| Frequency band | Scalp area | M | SD | M | SD | t | p |
| Eyes-closed Beta | Central | 0.932 | 0.370 | 0.864 | 0.421 | 0.861 | .392 |
|  | Frontal | 1.230 | 0.401 | 1.107 | 0.371 | 1.556 | .124 |
|  | Temporal | 1.356 | 0.539 | 1.280 | 0.547 | 0.689 | .493 |
|  | Parietal | 1.332 | 0.392 | 1.269 | 0.452 | 0.750 | .455 |
|  | Occipital | 1.797 | 0.571 | 1.690 | 0.552 | 0.933 | .354 |
| Eyes-open Alpha | Central | 1.895 | 0.705 | 1.624 | 0.744 | 1.746 | **.085^+^** |
|  | Frontal | 1.545 | 0.534 | 1.333 | 0.627 | 1.731 | **.088^+^** |
|  | Temporal | 2.142 | 0.709 | 1.994 | 0.815 | 0.916 | .363 |
|  | Parietal | 2.341 | 0.695 | 2.172 | 0.864 | 1.030 | .306 |
|  | Occipital | 2.555 | 0.775 | 2.523 | 0.905 | 0.180 | .858 |
| Eyes-open Beta | Central | 0.882 | 0.474 | 0.908 | 0.416 | -0.266 | .792 |
|  | Frontal | 1.461 | 0.600 | 1.377 | 0.536 | 0.672 | .504 |
|  | Temporal | 1.230 | 0.527 | 1.431 | 0.560 | -1.720 | **.090^+^** |
|  | Parietal | 1.152 | 0.450 | 1.215 | 0.403 | -0.670 | .505 |
|  | Occipital | 1.551 | 0.645 | 1.586 | 0.408 | -0.278 | .782 |

**Notes:** ^+^ marginally significant.

**Supplementary Material 3**

Group comparison of the resting-state MST metrics.

| Frequency band | Scalp area | TD |  | DD |  | Stats |  |
| --- | --- | --- | --- | --- | --- | --- | --- |
|  |  | M | SD | M | SD | t | p |
| Eyes-closed alpha | Degree | 1.725 | .075 | 1.716 | .063 | 0.648 | .519 |
|  | Kappa | 0.388 | .114 | 0.370 | .098 | 0.797 | .428 |
|  | BC | -1.963 | .029 | -1.958 | .028 | -0.886 | .378 |
|  | Diameter | -0.996 | .201 | -1.054 | .201 | 1.409 | .163 |
|  | Ecc | -1.301 | .202 | -1.361 | .200 | 1.482 | .142 |
|  | LF | -0.770 | .030 | -0.771 | .026 | 0.166 | .869 |
|  | Th | 2.394 | .025 | 2.398 | .022 | -0.791 | .431 |
| Eyes-closed beta | Degree | 1.593 | .028 | 1.596 | .028 | -0.498 | .620 |
|  | Kappa | 0.177 | .033 | 0.179 | .046 | -0.255 | .799 |
|  | BC | -1.912 | .018 | -1.916 | .018 | 1.102 | .273 |
|  | Diameter | -2.138 | .044 | -2.146 | .055 | 0.781 | .436 |
|  | Ecc | -2.435 | .043 | -2.443 | .055 | 0.736 | .464 |
|  | LF | -0.830 | .016 | -0.827 | .022 | -0.564 | .574 |
|  | Th | 2.357 | .018 | 2.359 | .026 | -0.273 | .786 |
| Eyes-open alpha | Degree | 1.677 | .042 | 1.676 | .064 | 0.072 | .943 |
|  | Kappa | 0.308 | .064 | 0.313 | .100 | -0.297 | .767 |
|  | BC | -1.949 | .017 | -1.948 | .027 | -0.146 | .884 |
|  | Diameter | -1.210 | .131 | -1.190 | .173 | -0.636 | .527 |
|  | Ecc | -1.515 | .131 | -1.496 | .172 | -0.621 | .536 |
|  | LF | -0.787 | .024 | -0.786 | .031 | -0.145 | .885 |
|  | Th | 2.385 | .026 | 2.389 | .029 | -0.675 | .502 |

**Notes:** Degree = maximum nodal degree; BC = betweenness centrality; Ecc = eccentricity; LF = leaf fraction; Th = tree hierarchy. Bold text represents significant effects; ^*^ represents significant results p < 0.05.

**Supplementary Material 4**

**4.1 Group comparison of the resting-state spectral power in Delta and Theta bands.**

| Frequency band | Scalp area | TD |  | DD |  | Stats | |
| --- | --- | --- | --- | --- | --- | --- | --- |
|  |  | M | SD | M | SD | t | p |
| Eyes-closed Delta | Central | 2.75 | 0.353 | 2.74 | 0.319 | 0.083 | .934 |
|  | Frontal | 3.26 | 0.246 | 3.33 | 0.299 | -1.324 | .189 |
|  | Temporal | 3.12 | 0.330 | 3.14 | 0.390 | -0.302 | .763 |
|  | Parietal | 3.21 | 0.341 | 3.21 | 0.375 | -0.046 | .963 |
|  | Occipital | 3.54 | 0.429 | 3.42 | 0.412 | 1.360 | .178 |
| Eyes-closed Theta | Central | 1.92 | 0.530 | 1.84 | 0.514 | 0.751 | .455 |
|  | Frontal | 1.83 | 0.442 | 1.82 | 0.385 | 0.062 | .951 |
|  | Temporal | 2.13 | 0.533 | 2.09 | 0.573 | 0.321 | .749 |
|  | Parietal | 2.36 | 0.566 | 2.27 | 0.605 | 0.766 | .446 |
|  | Occipital | 2.53 | 0.625 | 2.36 | 0.563 | 1.418 | .160 |
| Eyes-open Delta | Central | 2.72 | 0.403 | 2.75 | 0.303 | -0.485 | .630 |
|  | Frontal | 3.12 | 0.294 | 3.09 | 0.313 | 0.466 | .642 |
|  | Temporal | 3.06 | 0.416 | 3.11 | 0.289 | -0.551 | .584 |
|  | Parietal | 3.11 | 0.368 | 3.16 | 0.314 | -0.677 | .501 |
|  | Occipital | 3.38 | 0.436 | 3.4 | 0.355 | -0.221 | .826 |
| Eyes-open Theta | Central | 1.74 | 0.560 | 1.66 | 0.455 | 0.703 | .485 |
|  | Frontal | 1.75 | 0.433 | 1.63 | 0.379 | 1.281 | .205 |
|  | Temporal | 1.84 | 0.531 | 1.84 | 0.473 | 0.001 | .999 |
|  | Parietal | 1.96 | 0.557 | 1.94 | 0.516 | 0.167 | .868 |
|  | Occipital | 2.06 | 0.591 | 2.12 | 0.508 | -0.508 | .613 |

**4.2 Group comparison of the resting-state MST metrics in Delta and Theta bands.**

| Frequency band | MST | TD |  | DD |  | Stats |  |
| --- | --- | --- | --- | --- | --- | --- | --- |
|  | Metrics | M | SD | M | SD | t | p |
| Eyes-closed Delta | degree | 1.66 | 0.039 | 1.65 | 0.039 | 0.161 | .873 |
|  | Kappa | 0.276 | 0.055 | 0.28 | 0.060 | 0.016 | .987 |
|  | BC | -1.94 | 0.023 | -1.94 | 0.026 | 0.070 | .945 |
|  | Diameter | -1.29 | 0.067 | -1.27 | 0.081 | -1.261 | .211 |
|  | Ecc | -1.59 | 0.069 | -1.57 | 0.081 | -1.072 | .286 |
|  | LF | -0.80 | 0.019 | -0.80 | 0.023 | 0.357 | .722 |
|  | Th | 2.38 | 0.018 | 2.38 | 0.021 | 0.726 | .470 |
| Eyes-closed Theta | degree | 1.64 | 0.039 | 1.64 | 0.036 | 0.218 | .828 |
|  | Kappa | 0.25 | 0.055 | 0.24 | 0.057 | 0.433 | .666 |
|  | BC | -1.93 | 0.018 | -1.93 | 0.021 | 0.150 | .881 |
|  | Diameter | -1.44 | 0.061 | -1.43 | 0.067 | -0.860 | .392 |
|  | Ecc | -1.74 | 0.059 | -1.73 | 0.064 | -1.017 | .312 |
|  | LF | -0.81 | 0.019 | -0.81 | 0.021 | 0.412 | .681 |
|  | Th | 2.37 | 0.019 | 2.37 | 0.021 | 0.574 | .568 |
| Eyes-open Delta | degree | 1.64 | 0.040 | 1.65 | 0.038 | -0.747 | .458 |
|  | Kappa | 0.25 | 0.059 | 0.26 | 0.056 | -0.645 | .521 |
|  | BC | -1.93 | 0.021 | -1.94 | 0.019 | 0.521 | .604 |
|  | Diameter | -1.30 | 0.063 | -1.28 | 0.084 | -1.160 | .249 |
|  | Ecc | -1.60 | 0.060 | -1.58 | 0.084 | -1.230 | .223 |
|  | LF | -0.81 | 0.022 | -0.81 | 0.021 | -0.588 | .559 |
|  | Th | 2.37 | 0.026 | 2.37 | 0.024 | -0.673 | .504 |
| Eyes-open Theta | degree | 1.62 | 0.039 | 1.62 | 0.033 | 0.454 | .652 |
|  | Kappa | 0.23 | 0.050 | 0.22 | 0.051 | 0.569 | .571 |
|  | BC | -1.93 | 0.023 | -1.93 | 0.024 | -0.765 | .447 |
|  | Diameter | -1.45 | 0.078 | -1.45 | 0.053 | -0.207 | .837 |
|  | Ecc | -1.75 | 0.076 | -1.75 | 0.052 | -0.073 | .942 |
|  | LF | -0.82 | 0.022 | -0.82 | 0.023 | 0.554 | .582 |
|  | Th | 2.37 | 0.021 | 2.37 | 0.024 | 0.334 | .740 |

**Supplementary Material 5**

**5.1 Group comparison of the one-back spectral power in Delta and Theta bands.**

| Frequency band | Scalp area | Group | | | Condition | | | Condition × Group | | |
| --- | --- | --- | --- | --- | --- | --- | --- | --- | --- | --- |
|  |  | F _(1, 113)_ | p | η^​2^ | F _(1, 113)_ | p | η​^2^ | F _(1, 113)_ | p | η​^2^ |
| One-back Delta | central | 0.362 | .549 | .003 | **19.187** | **<.001***** | **.145** | 0.205 | .651 | .002 |
|  | frontal | 0.139 | .710 | .001 | 2.433 | .122 | .021 | 0.177 | .675 | .002 |
|  | temporal | 2.532 | .114 | .022 | 3.898 | .051 | .033 | 0.027 | .870 | <.001 |
|  | parietal | 0.958 | .330 | .008 | **7.130** | **.009**** | **.059** | 0.018 | .895 | <.001 |
|  | occipital | 0.102 | .750 | .001 | **6.270** | **.014**** | **.053** | 0.020 | .887 | <.001 |
| One-back Theta | central | 0.418 | .519 | .004 | **9.697** | **.002**** | **.079** | 2.112 | .149 | .018 |
|  | frontal | 1.651 | .202 | .014 | **10.900** | **.001**** | **.088** | 2.301 | .132 | .020 |
|  | temporal | 0.003 | .954 | <.001 | **38.428** | **<.001***** | **.254** | 0.150 | .699 | .001 |
|  | parietal | 0.011 | .917 | <.001 | **83.320** | **<.001***** | **.424** | 0.007 | .934 | <.001 |
|  | occipital | 0.779 | .379 | .007 | **65.882** | **<.001***** | **.368** | 0.090 | .764 | .001 |

**Note.** Bold text indicates significant effects. Significance markers: * p < .05, ** p < .01, *** p < .001.

**5.2 Group comparison of the one-back MST metrics in Delta and Theta bands.**

| Frequency | MST | Group |  |  | Condition | |  | Condition × Group | | |
| --- | --- | --- | --- | --- | --- | --- | --- | --- | --- | --- |
| band | Metrics | F _(1, 113)_ | p | η​^2^ | F _(1, 113)_ | p | η​^2^ | F _(1, 113)_ | p | η​^2^ |
| One-back Delta | degree | 0.066 | .797 | .001 | 2.105 | .150 | .018 | 0.141 | .708 | .001 |
|  | kappa | 0.215 | .644 | .002 | 0.605 | .438 | .005 | 0.451 | .503 | .004 |
|  | BC | 2.988 | .087 | .026 | 0.405 | .526 | .004 | 0.415 | .521 | .004 |
|  | diameter | 0.002 | .968 | <.001 | **4.905** | **.029*** | **.042** | 0.006 | .939 | <.001 |
|  | Ecc | 0.006 | .937 | <.001 | **4.127** | **.045*** | **.035** | 0.004 | .951 | <.001 |
|  | LF | 2.030 | .157 | .018 | 0.022 | .882 | <.001 | 0.495 | .483 | .004 |
|  | Th | 2.744 | .100 | .024 | 0.195 | .659 | .002 | 2.069 | .153 | .018 |
| One-back Theta | degree | 0.921 | .339 | .008 | 0.435 | .511 | .004 | 1.141 | .288 | .010 |
|  | kappa | 1.535 | .218 | .013 | 0.593 | .443 | .005 | 0.947 | .332 | .008 |
|  | BC | 0.719 | .397 | .003 | 0.261 | .610 | .001 | 0.273 | .602 | .001 |
|  | diameter | 1.298 | .257 | .011 | 0.162 | .688 | .001 | 0.339 | .562 | .003 |
|  | Ecc | 1.491 | .225 | .013 | 0.242 | .624 | .002 | 0.291 | .590 | .003 |
|  | LF | 0.351 | .555 | .003 | 0.160 | .690 | .001 | 0.566 | .453 | .005 |
|  | Th | 0.671 | .415 | .006 | 0.239 | .626 | .002 | 0.654 | .420 | .006 |

**Note.** Bold text indicates significant effects. Significance markers: * p < .05, ** p < .01, *** p < .001.

**Supplementary Material 6**

**6.1 MST plots for eyes‑closed alpha and beta bands and eyes-open alpha band**

**
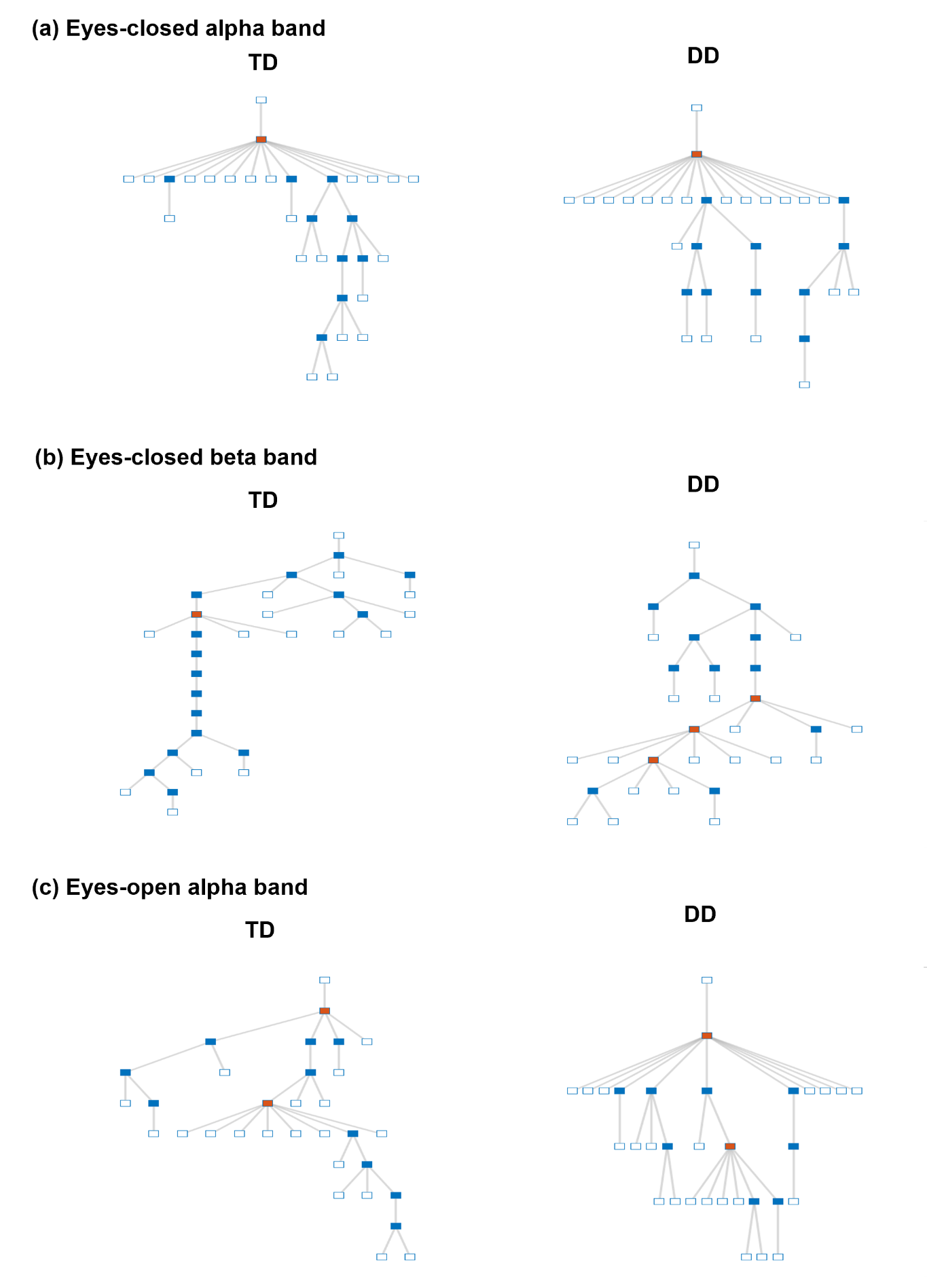
**

**6.2 MST plots for one-back beta band**

**
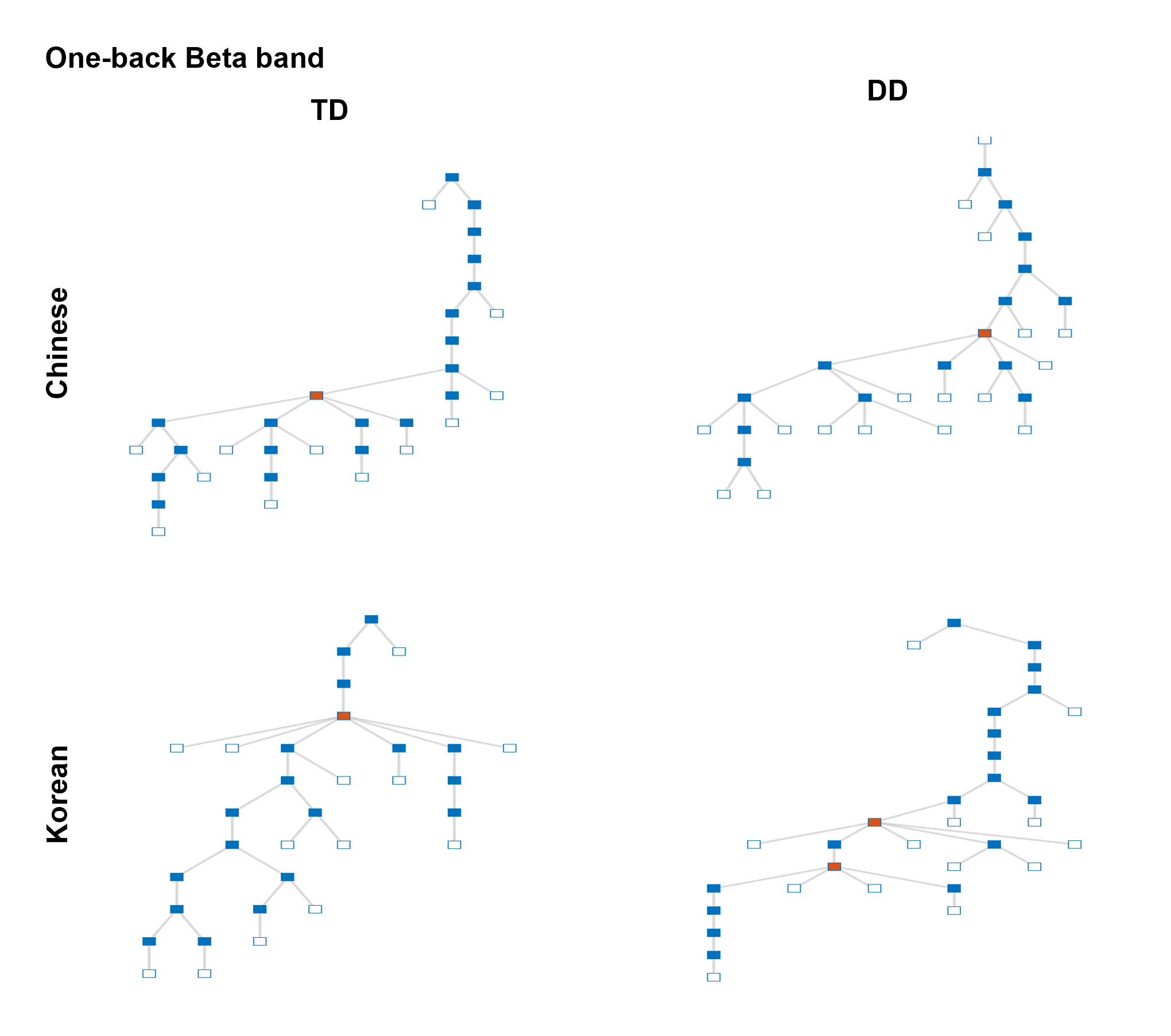
**

**Supplementary material 7**

Correlation results between resting-state Beta-band MST metrics and reading measurements within the DD group.

| Metric | Measure | Correlation | p_value |
| --- | --- | --- | --- |
| Beta_degree | Reading_acc | .042 | .761 |
| Beta_degree | Reading_fluency | .037 | .786 |
| Beta_kappa | Reading_acc | .181 | .185 |
| Beta_kappa | Reading_fluency | **.292** | **.030*** |
| Beta_BC | Reading_acc | -.140 | .309 |
| Beta_BC | Reading_fluency | -.182 | .183 |
| Beta_LF | Reading_acc | .200 | .143 |
| Beta_LF | Reading_fluency | **.297** | **.028*** |

**Notes:** * p < 0.05.

**Supplementary material 8**

Correlation results between Chinese-Korean one-back task Alpha-band MST metrics and reading measurements within the DD group.

| Metric | Measure | Correlation | p_value |
| --- | --- | --- | --- |
| Alpha_degree_c | Reading_acc | .040 | .759 |
| Alpha_degree_c | Reading_fluency | .110 | .397 |
| Alpha_degree_c | dprime_c | .141 | .277 |
| Alpha_degree_c | dprime_k | .111 | .392 |
| Alpha_diameter_c | Reading_acc | .022 | .865 |
| Alpha_diameter_c | Reading_fluency | .039 | .763 |
| Alpha_diameter_c | dprime_c | -.089 | .494 |
| Alpha_diameter_c | dprime_k | -.102 | .434 |
| Alpha_ecc_c | Reading_acc | .008 | .949 |
| Alpha_ecc_c | Reading_fluency | .026 | .843 |
| Alpha_ecc_c | dprime_c | -.074 | .572 |
| Alpha_ecc_c | dprime_k | -.090 | .489 |
| Alpha_degree_k | Reading_acc | .003 | .981 |
| Alpha_degree_k | Reading_fluency | .014 | .916 |
| Alpha_degree_k | dprime_c | .166 | .201 |
| Alpha_degree_k | dprime_k | **-.266** | **.038*** |
| Alpha_diameter_k | Reading_acc | .091 | .485 |
| Alpha_diameter_k | Reading_fluency | .101 | .440 |
| Alpha_diameter_k | dprime_c | -.020 | .880 |
| Alpha_diameter_k | dprime_k | -.121 | .353 |
| Alpha_Ecc_k | Reading_acc | .102 | .435 |
| Alpha_Ecc_k | Reading_fluency | .114 | .380 |
| Alpha_Ecc_k | dprime_c | -.026 | .845 |
| Alpha_Ecc_k | dprime_k | -.120 | .355 |

**Notes:** * p < 0.05.
